# Bog plant/lichen tissue nitrogen and sulfur concentrations as indicators of emissions from oil sands development in Alberta, Canada

**DOI:** 10.1007/s10661-021-08929-y

**Published:** 2021-03-23

**Authors:** R. Kelman Wieder, Melanie A. Vile, Kimberli D. Scott, Cara M. Albright, James C. Quinn, Dale H. Vitt

**Affiliations:** 1 Department of Biology, Villanova University, Villanova, PA 19085 USA; 2 Center for Biodiversity and Ecosystem Stewardship, Villanova University, Villanova, PA 19085 USA; 3 Faculty of Science and Technology, Athabasca University, Athabasca, Alberta T9S 3A3 Canada; 4 Department of Health, West Chester University, West Chester, PA 19383 USA; 5 School of Biological Sciences, Southern Illinois University, Carbondale, IL 62901 USA

**Keywords:** Bryophyte, Monitoring, Peatland, *Sphagnum*

## Abstract

Increasing gaseous emissions of nitrogen (N) and sulfur (S) associated with oil sands development in northern Alberta (Canada) has led to changing regional wet and dry N and S deposition regimes. We assessed the potential for using bog plant/lichen tissue chemistry (N and S concentrations, C:N and C:S ratios, in 10 plant/lichen species) to monitor changing atmospheric N and S deposition through sampling at five bog sites, 3–6 times per growing season from 2009 to 2016. During this 8-year period, oil sands N emissions steadily increased, while S emissions steadily decreased. We examined the following: (1) whether each species showed changes in tissue chemistry with increasing distance from the Syncrude and Suncor upgrader stacks (the two largest point sources of N and S emissions); (2) whether tissue chemistry changed over the 8 year period in ways that were consistent with increasing N and decreasing S emissions from oil sands facilities; and (3) whether tissue chemistry was correlated with growing season wet deposition of NH_4_^+^-N, NO_3_^−^-N, or SO_4_^2−^-S. Based on these criteria, the best biomonitors of a changing N deposition regime were *Evernia mesomorpha*, *Sphagnum fuscum*, and *Vaccinium oxycoccos*. The best biomonitors of a changing S deposition regime were *Evernia mesomorpha*, *Cladonia mitis*, *Sphagnum fuscum*, *Sphagnum capillifolium*, *Vaccinium oxycoccos*, and *Picea mariana*. Changing N and S deposition regimes in the oil sands region appear to be influencing N and S cycling in what once were pristine ombrotrophic bogs, to the extent that these bogs may effectively monitor future spatial and temporal patterns of deposition.

**Supplementary Information:**

The online version contains supplementary material available at 10.1007/s10661-021-08929-y.

## Introduction

Indigenous peoples of northern Alberta had known about the existence of bitumen associated with oil sands long before Peter Pond’s first written account in 1778 (Hein, 2000). Sun Oil Company invested $240 million to build the Great Canadian Oil Sands facility, where an open pit mine and an oil upgrader began producing 45,000 bbl day^−1^ in 1967. In 2001, Cenovus Energy’s Foster Creek plant became the first in situ oil sands operation, using SAGD (steam-assisted gravity drainage) technology (CAPP, 2019). Oil sands development has steadily increased over time with total oil sands production reaching 171,084,241 m^3^ (1.1 billion bbl) in 2019 (AER, 2020). Most of the oil produced from the oil sands region is exported to the USA, and since 2009, the USA has imported more oil from Canada than from any other country (US EIA, 2020).

**Fig. 1:**
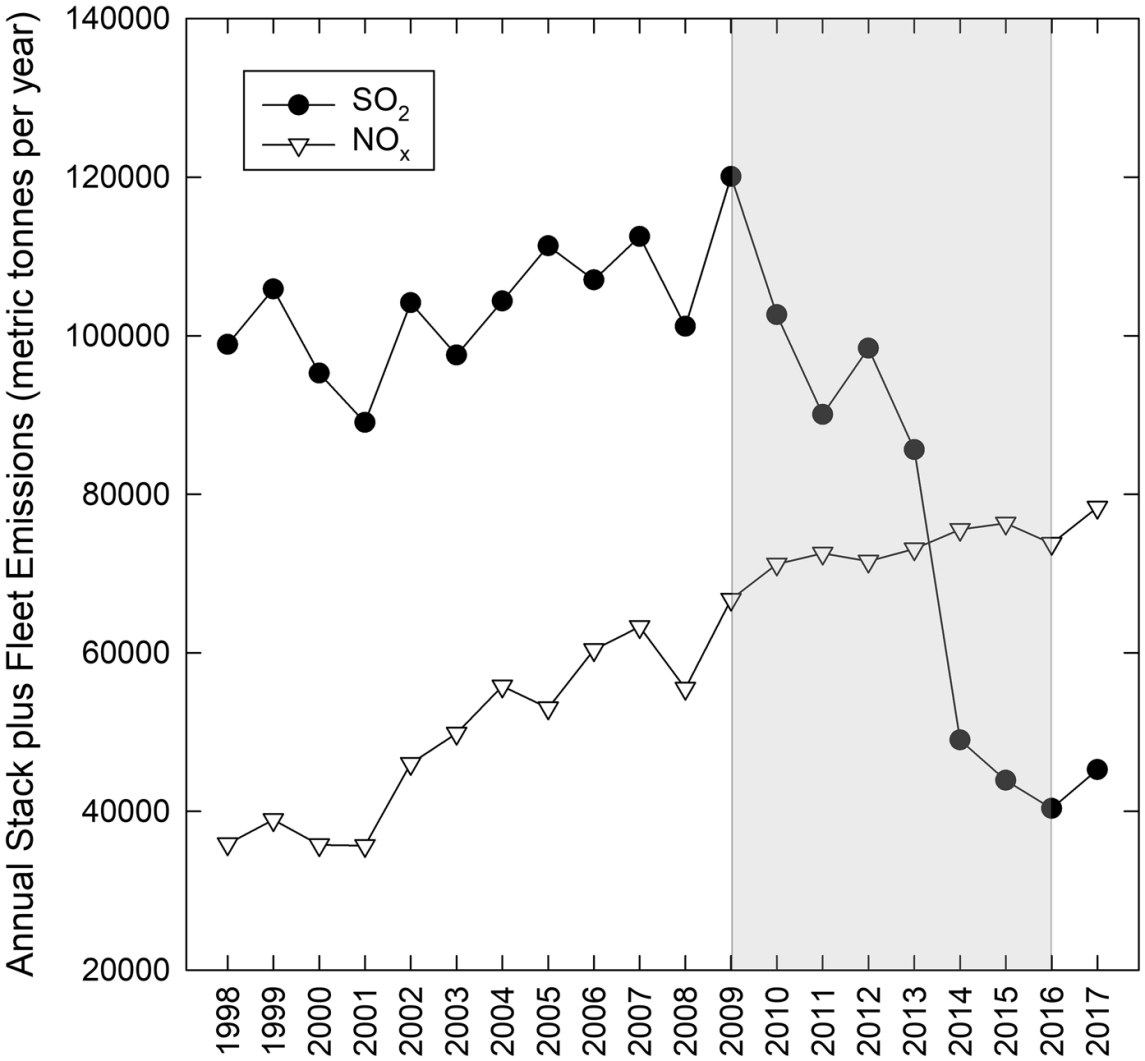
Combined upgrader stack and mine fleet emissions of NO_*x*_ and SO_2_ and for Syncrude Mildred Lake, Suncor Aurora, Suncor Base Plant, Suncor Firebag, Albian Sands Muskeg River/Jackpine, CNRL Horizon, and Nexen Long Lake facilities. Stack emissions were obtained from Canada’s National Pollutant Release Inventory (NPRI) database. Annual emissions of NO_*x*_ and SO_2_ from oil sands facility fleet vehicles were calculated from published emission factors (0.780 kg NO_*x*_ per m^3^ of crude bitumen production; 0.015 kg SO_2_ per m^3^ of crude bitumen production; CEMA, 2012)

Associated with oil sands development is the release of gaseous N and S compounds into the atmosphere, both from upgrader stacks and diesel fuel–powered mine fleets (Davidson & Spink, 2018). Over the past 20 years, N emissions from oil sands operations have steadily increased, while S emissions peaked in 2009 and have been declining since (Fig. 1). These gaseous N and S emissions ultimately are deposited on the region’s natural ecosystems in both wet deposition (as NH_4_^+^-N, NO_3_^−^-N, and SO_4_^2−^-S; Fenn et al., 2015; Wieder et al., 2016a, 2016b) and dry deposition (mainly as NH_3_, NO_2_, HNO_3_/HONO, and SO_2_; Hsu et al., 2016). Across the 140,329-km^2^ Oil Sands Administrative Area, bogs cover 8,962 km^2^ and fens cover 29,083 km^2^ (Wieder et al., 2016a). Given the ombrotrophic nature of bogs, they are likely to be especially susceptible to changing atmospheric N and/or S deposition regimes. Synoptic sampling of 23 bogs within a 3,255-km^2^ area in the oil sands region revealed spatial gradients in N and S concentrations in the tissues of some lichen, moss, and vascular plant species that were correlated with regional gradients in NH_4_^+^-N, NO_3_^−^-N, and SO_4_^2−^-S deposition (Wieder et al., 2016a). Experimental field addition of N (as NH_4_NO_3_ in simulated rainfall) to an Alberta bog over 5 years also resulted in increased N concentrations in tissues of some plant species, as well as other structural and functional responses (Wieder et al., 2019). Increasing tissue N and S concentrations are an indication of uptake in excess of plant demands for growth, and especially for N, may cause plant stress (Marschner, 2012; Rennenberg, 1984).

Here we report eight years of monitoring of plant and lichen N and S concentrations in five peatlands located at different distances from oil sands bitumen production, upgrading, and supporting/reclamation operations. Our major objective was to assess whether plant/lichen tissue N and/or S concentrations can serve as effective biomonitors of changing atmospheric N and S deposition regimes. Toward this end, we examined temporal (within- and between-years) patterns in N and S concentrations in 10 bog plant/lichen species at 5 peatland sites. We used three criteria to assess the suitability of plant/lichen tissue N and S concentrations as biomonitors: (1) whether a species showed increasing C:N and/or C:S ratios with increasing distance from the Syncrude and Suncor upgrader stacks (the two largest point sources of N and S emissions), assessed using both ANOVA and regression approaches; (2) whether tissue C:N decreased or C:S ratios increased over the 8 year period; and (3) whether tissue C:N and/or C:S ratios were correlated with growing season wet deposition of NH_4_^+^-N, NO_3_^−^-N, or SO_4_^2−^-S. A secondary objective was to evaluate whether N and S emissions from oil sands development are affecting plant/lichen tissue N and S concentrations in ways that could have implications for bog ecosystem structure and function.

## Materials and methods

### Study sites

**Fig. 2:**
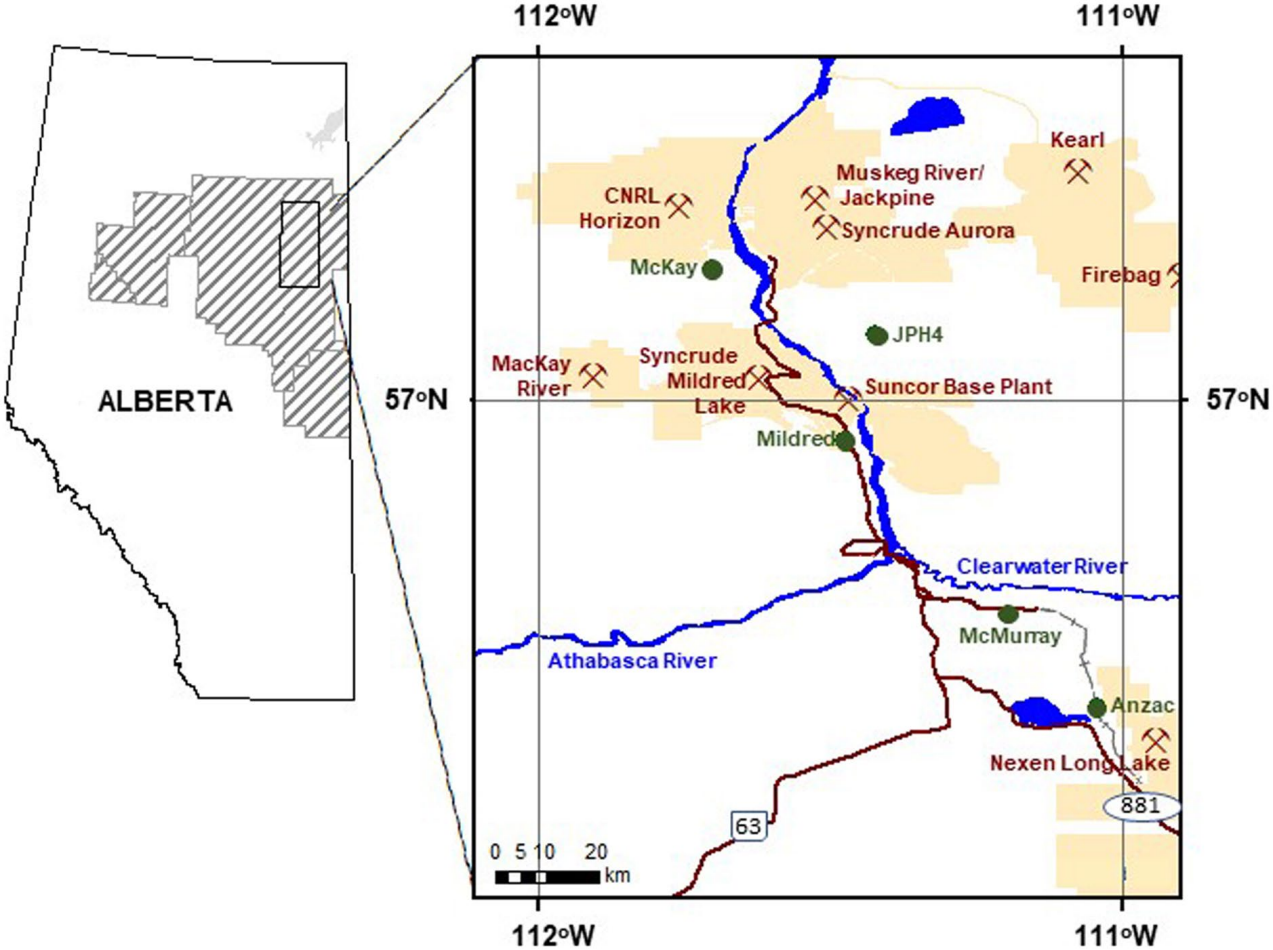
Map of the oil sands region showing oil sands facilities (mine symbols surrounded by lease areas) and the five bog monitoring sites (green circles)

We report data from five peatlands (Mildred, 56° 55′ 49″ N, 111° 28′ 31″ W; JPH4, 57° 6′ 45″ N, 111° 25′ 23″ W; McKay, 57° 13′ 41″ N, 111° 42′ 11″ W; McMurray, 56° 37′ 37″ N, 111° 11′ 44″ W; Anzac, 56° 28′ 8″ N, 111° 2′ 34″ W) at different distances from oil sands mining operations north of Fort McMurray Canada (Fig. 2). Distances from the midpoint between the Syncrude and Suncor upgrader stacks are 11, 12, 24, 49, and 69 km for Mildred, JPH4, McKay, McMurray, and Anzac, respectively (Wieder et al., 2016b). Four of the sites are ombrotrophic bogs, with a nearly continuous cover of *Sphagnum* mosses (predominantly *Sphagnum fuscum*, with some *Sphagnum capillifolium*, *Sphagnum angustifolium*, and *Sphagnum magellanicum*), an abundance of ericaceous shrubs (*Rhododendron groenlandicum*, *Vaccinium oxycoccos*, *Vaccinium vitis-idaea*), and acidic porewaters (Wieder et al., 2016b). Mildred is best described as a mixed mire with minerogenous water affecting lawns and carpets (circumneutral pore water pH) and ombrogenous hummocks dominated by *S. fuscum* (Wieder et al., 2016b). Despite having porewater with a higher pH than is typical of Alberta bogs, Mildred was chosen because of vegetational similarity to bogs and its close proximity to the Syncrude Mildred Lake and Suncor base plant upgrader stacks.

### Sampling and analysis

Beginning in 2009, plant/lichen tissue sampling occurred at Mildred, McKay, McMurray, and Anzac peatlands, with 5–6 sampling dates per year between early May and mid-October. In 2010, we added a fifth site (JPH4) when construction of the East Athabasca highway allowed site access to a previously roadless region. The Wood Buffalo Environmental Association instructed us to decommission the Mildred site at the end of the 2012 sampling season, as the site was expected to be overrun by new construction at the AOSTRA Road interchange on Highway 63. Upon discovering that the Mildred site was not affected by highway construction, we reestablished the site and resumed sampling in 2015. However, the Mildred site was completely burned in the 2016 Fort McMurray wildfire. Noncontinuation of funding resulted in a lower temporal sampling intensity (3 sampling dates per year in June, July, August) in 2013–2016 than in previous years.

On each sampling date at each site, we collected five replicate samples of the mosses *Sphagnum fuscum* and *Sphagnum capillifolium* (about 100 cm^2^ of surface moss; capitula retained for analysis), the lichens *Evernia mesomorpha* (whole lichen thalli) and *Cladonia mitis* (top 2.5 cm of thalli), the ericaceous shrubs *Rhododendron groenlandicum* (topmost 3–5 leaves from individual plants all from current year’s growth), *Vaccinium vitis-idaea* (topmost 3–5 leaves from individual plants all from current year’s growth), *Vaccinium oxycoccos* (aboveground portions of plant with leaves separated from stems; leaves retained for analysis), the deciduous forbs *Rubus chamaemorus* and *Maianthemum trifolium* (current year leaves), and the dominant tree *Picea mariana* (apical shoots of current year’s growth).

In the laboratory, samples were cleaned to remove debris, oven-dried (60 °C), and ground in a Wiley micromill; each replicate plant sample yielded 2–10 g of dried ground material. One subsample from each replicate, ground sample was analyzed for total C, N, and S concentrations (mg g^−1^) on a Leco Truspec CNS analyzer. Standard curves were constructed using differing quantities (0.1–0.2 g) of standard reference materials (for C, NIST 1632b coal, Leco 1018 tobacco leaves; for N, NIST 1547 peach leaves, NIST 1632b coal, Leco 1018 tobacco leaves; for S, NIST 1632b coal, Leco 1018 tobacco leaves). All standard curves had *R*^2^ values > 0.999. Analyses with 0.2 g of samples had limits of detection for C, N, and S of 12.4, 0.29, and 0.15 mg g^−1^, respectively. Various standard plant tissues were analyzed to assess precision and accuracy. Coefficients of variation for N and S measurements averaged 1.8, 3.3, and 2.6%, respectively. On average, mean measured C, N, and S concentrations were within 0.1, 1.5, and 2.5% of certified values, respectively (Table S1).

### Statistical analyses

Tissue C, N, and S concentrations, as well as C:N, C:S, and N:S ratios were not normally distributed (Kolmogorov–Smirnov test, *p* < 0.01). Therefore, for these six variables, we examined site differences, blocking on sampling date, using the nonparametric Friedman’s test, with *a posteriori* site median comparisons using Tukey’s Honestly Significant Difference Test (Pereira et al., 2015). To examine whether tissue N or C:S ratios increased over the 8 years of the study, or whether tissue S concentrations or C:N ratios decreased over time, we used non-parametric correlations (Kendall’s Tau, one-sided tests). We also used Kendall’s Tau correlations (one-sided tests) to examine whether tissue N concentrations were positively correlated and C:N concentrations were negatively correlated with growing season NH_4_^+^-N and/or NO_3_^−^-N deposition and whether tissue S concentrations were positively correlated and C:S concentrations were negatively correlated with growing season SO_4_^2−^-S deposition (deposition quantified from ion exchange resin collectors; Wieder et al., 2016b).

Finally, we used nonlinear regression to examine whether tissue N and S concentrations decreased and whether tissue C:N and C:S ratios increased exponentially with distance from the main sources of N and S emissions, the Syncrude Mildred Lake and Suncor Base Plant upgrader stacks. The equation for decreases in plant/lichen tissue N or S with distance was as follows:

$$\mathrm{Tissue}\;\mathrm{N}\;\mathrm{or} \;\mathrm S\;\left(\mathrm{mg}\;\mathrm g^{-1}\right)=A+B\times e^{-(\mathrm C\times\mathrm{Distance})}$$

The equation for increases in plant/lichen tissue C:N or C:S with distance was as follows:

$$\mathrm{Tissue}\;\mathrm C:\mathrm{N}\;\mathrm{or}\;\mathrm C:\mathrm{S}\;=A+B\times(1-e^{-\left(\mathrm C\times\mathrm{Distance}\right)})$$

Regression analyses were carried out using PROC NLIN in SAS (v 9.4).

### Biomonitoring potential

We assessed the potential of each plant/lichen species as a biomonitor of changing atmospheric N and S deposition regimes using four criteria: (1) did tissue C:N or C:S ratios increase with distance from midpoint between Syncrude and Suncor stacks, assessed using Friedman’s test; (2) did tissue C:N or C:S ratios increase with distance from midpoint between Syncrude and Suncor stacks, assessed using exponential regression; (3) were changes in tissue C:N or C:S ratios over time consistent with increasing N and decreasing S emissions from oil sands facilities; and (4) was tissue C:N inversely correlated with growing season NH_4_^+^-N and/or NO_3_^−^-N deposition and was tissue C:S inversely correlated with growing season SO_4_^2−^-S deposition.

### Power analysis

We conducted power analyses to determine the number of samples required per site on each sampling date to detect a 20% difference in tissue N or S concentration, C:N, or C:S ratio between two sites or two sampling dates at a *p* level of 0.10 and a power ≥ 0.80. Power analyses were also conducted to calculate the power to detect a 20% difference in tissue N or S concentration, C:N, or C:S ratio between two sites or two sampling dates at a *p* level of 0.10 and a sample size of 5, as we used throughout this study. Power analyses were carried out using PROC POWER in SAS (v. 9.4) using the pooled within-group standard deviation for each species across all sampling dates.

### Influence of different temporal sampling intensities

We examined how less intensive sampling would have affected the ability to detect differences in plant/lichen tissue chemistry (Friedman’s test), exponential regressions of tissue chemistry as a function of distance from the Syncrude and Suncor upgrader stacks, changes in tissue chemistry over time (Kendall's Tau), and correlations between plant/lichen tissue chemistry with growing season atmospheric deposition of NH_4_^+^-N, NO_3_^−^-N, or SO_4_^2−^-S (Kendall’s Tau). Using subsets of our full data set, two sampling schemes were examined: one sampling date per year between August 5 and August 9 and one sampling date between August 5 and August 9 in 2010, 2013, and 2015 (years in which we had data for all five sites).

## Results

**Fig. 3:**
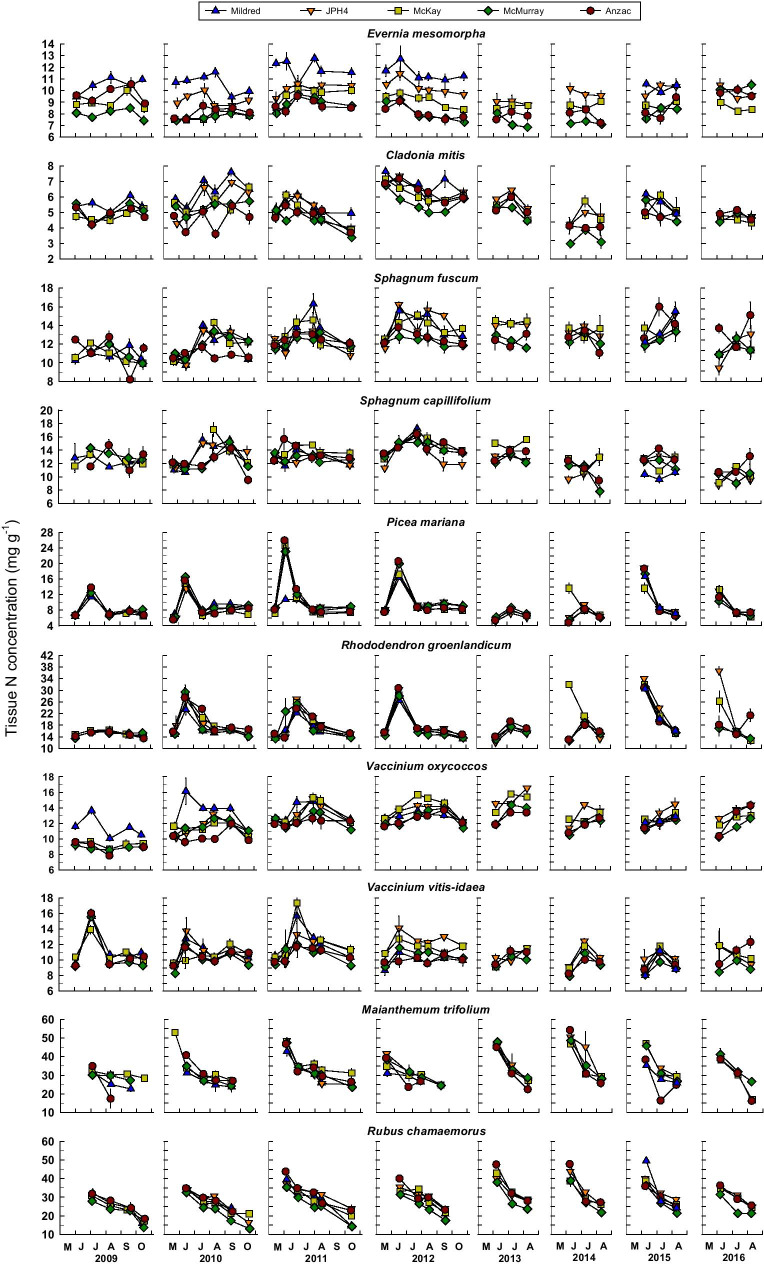
Temporal changes in N concentrations (mg g^−1^) in lichen thalli, *Sphagnum* capitula, and plant leaves/needles at the five bog sites. Values are means ± standard errors, *n* = 5

**Fig. 4:**
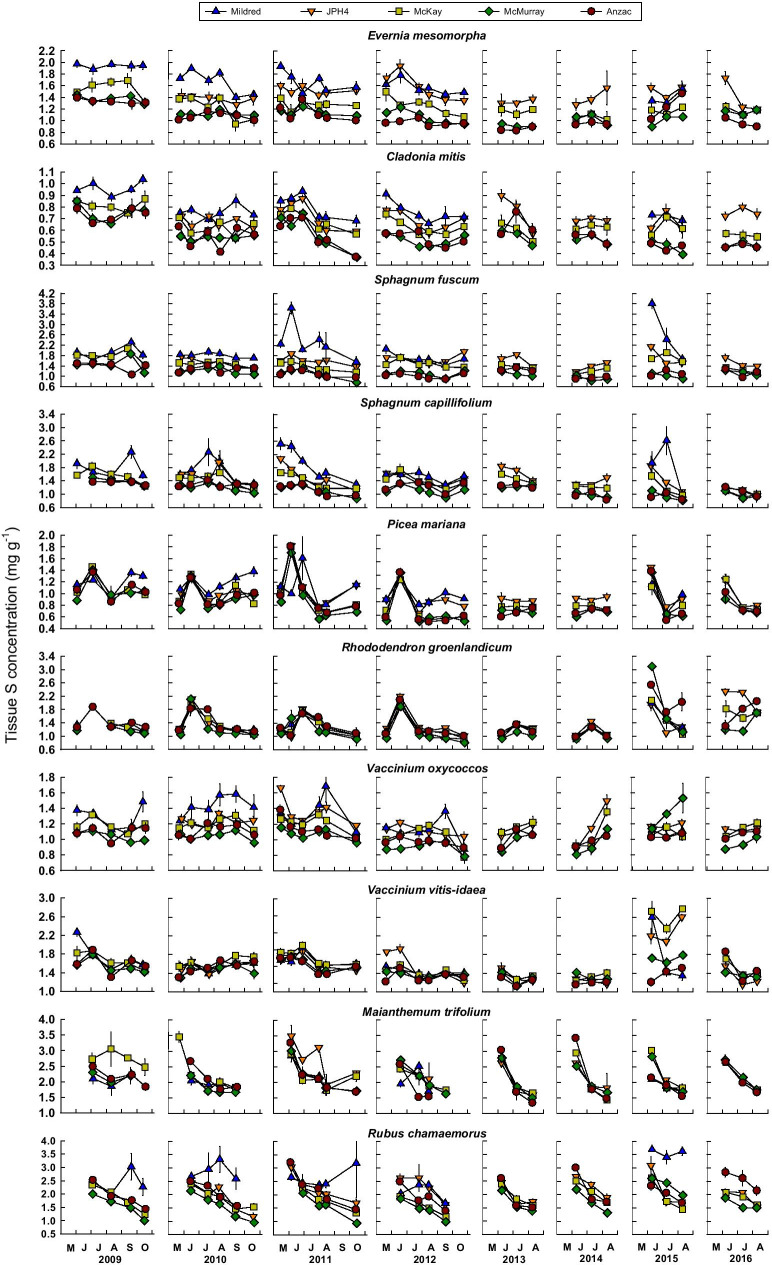
Temporal changes in S concentrations (mg g^−1^) in lichen thalli, *Sphagnum* capitula, and plant leaves/needles at the five bog sites. Values are means ± standard errors, *n* = 5

Median N and S concentrations as well as C:N, C:S, and N:S ratios in plant/lichen tissues averaged across all sampling dates, differed between sites for all 10 species (Table 1). For most of the species, we did not observe consistent within-year variability in plant/lichen tissue N or S concentrations (Figs. 3 and 4). However, for the two forbs, *M. trifolium* and *R. chamaemorus*, leaf N and S concentrations were highest at the beginning of the growing season and declined as the growing season progressed (Figs. 3 and 4). Further, *P. mariana* needles, *R. groenlandicum* leaves, and *V. vitis-idaea* leaves exhibited early season peaks in N concentrations in 2009–2012 (Fig. 3), accompanied by peaks in S concentration for *P. mariana* needles and *R. groenlandicum* leaves (Fig. 4), most likely representing retranslocation of stored N and S to support new leaf/needle growth. These sampling dates were excluded from analyses of changing N and S concentrations or C:N and C:S ratios with time and with distance from Syncrude and Suncor upgrader stacks.

**Table 1:** Median tissue concentrations of C, N, and S and C:N, C:S, and N:S ratios. For each parameter and each species, medians with the same letter superscript do not differ significantly (*p* < 0.05) per Friedman’s test (sampling date as the blocked factor); *a posteriori* comparisons made using Tukey’s Honestly Significant Difference Test (*α* = 0.05) (Pereira et al., 2015)

Species	Site	C (mg/g)	N (mg/g)	S (mg/g)	C:N	C:S	N:S
*Evernia mesomorpha*	Mildred	423 ^d^	10.9 ^a^	1.64 ^a^	38.3 ^e^	257 ^e^	6.9 ^c^
	JPH4	430 ^b^	9.7 ^b^	1.44 ^b^	43.8 ^d^	301 ^d^	6.8 ^d^
	McKay	427 ^c^	8.7 ^c^	1.26 ^c^	48.8 ^c^	337 ^c^	7.1 ^c^
	McMurray	454 ^a^	8.0 ^d^	1.11 ^d^	56.4 ^a^	408 ^b^	7.4 ^b^
	Anzac	454 ^a^	8.5 ^c^	1.04 ^d^	53.1 ^b^	437 ^a^	7.9 ^a^
*Cladonia mitis*	Mildred	441 ^b^	6.0 ^a^	0.79 ^a^	73.3 ^c^	548 ^d^	7.5 ^c^
	JPH4	435 ^b^	5.7 ^b^	0.69 ^b^	76.1 ^c^	617 ^c^	8.0 ^c^
	McKay	441 ^b^	5.2 ^c^	0.64 ^c^	83.0 ^b^	679 ^b^	8.2 ^b^
	McMurray	444 ^a^	5.0 ^d^	0.54 ^d^	89.2 ^a^	825 ^a^	9.5 ^a^
	Anzac	448 ^a^	5.1 ^cd^	0.56 ^d^	86.6 ^a^	798 ^a^	9.1 ^a^
*Sphagnum fuscum*	Mildred	432 ^e^	12.5 ^a^	1.83 ^a^	34.7 ^c^	243 ^d^	6.6 ^d^
	JPH4	442 ^d^	12.7 ^a^	1.56 ^b^	34.7 ^bc^	285 ^c^	8.2 ^c^
	McKay	453 ^c^	12.8 ^a^	1.43 ^c^	35.4 ^b^	318 ^b^	9.1 ^b^
	McMurray	459 ^b^	12.0 ^b^	1.13 ^d^	37.9 ^a^	413 ^a^	10.5 ^a^
	Anzac	464 ^a^	12.1 ^b^	1.14 ^d^	38.1 ^a^	407 ^a^	10.9 ^a^
*Sphagnum capillifolium*	Mildred	451 ^c^	13.1 ^ab^	1.58 ^a^	35.8 ^ab^	281 ^d^	8.0 ^d^
	JPH4	441 ^d^	12.6 ^b^	1.39 ^b^	35.3 ^ab^	319 ^c^	9.0 ^c^
	McKay	458 ^b^	13.2 ^a^	1.36 ^c^	34.8 ^b^	340 ^b^	9.7 ^b^
	McMurray	460 ^b^	12.7 ^b^	1.12 ^e^	36.1 ^a^	409 ^a^	11.2 ^a^
	Anzac	466 ^a^	12.7 ^ab^	1.20 ^d^	36.2 ^ab^	389 ^a^	11.0 ^a^
*Picea mariana*	Mildred	517 ^a^	8.1 ^a^	1.10 ^a^	59.7 ^bc^	467 ^c^	8.3 ^d^
	JPH4	511 ^c^	8.4 ^a^	0.94 ^a^	60.5 ^cd^	534 ^c^	9.2 ^d^
	McKay	510 ^bc^	7.4 ^c^	0.83 ^b^	68.7 ^a^	607 ^b^	9.2 ^c^
	McMurray	512 ^b^	8.2 ^a^	0.77 ^c^	61.9 ^d^	668 ^a^	11.2 ^a^
	Anzac	511 ^b^	7.8 ^b^	0.81 ^b^	66.4 ^b^	643 ^b^	10.1 ^b^
*Rhododendron groenlandicum*	Mildred	543 ^d^	15.7 ^b^	1.22 ^c^	34.7 ^a^	445 ^b^	13.2 ^b^
	JPH4	549 ^a^	16.1 ^a^	1.26 ^a^	33.8 ^b^	436 ^cd^	13.5 ^b^
	McKay	545 ^c^	15.7 ^a^	1.21 ^bc^	34.7 ^bc^	451 ^bc^	13.7 ^b^
	McMurray	547 ^bc^	15.3 ^b^	1.09 ^d^	35.8 ^a^	505 ^a^	14.5 ^a^
	Anzac	548 ^ab^	16.2 ^a^	1.26 ^ab^	33.5 ^c^	432 ^d^	13.5 ^b^
*Vaccinium oxycoccos*	Mildred	495 ^d^	12.8 ^a^	1.20 ^a^	39.1 ^b^	410 ^d^	10.5 ^b^
	JPH4	501 ^c^	13.0 ^ab^	1.18 ^a^	38.9 ^b^	426 ^c^	10.9 ^b^
	McKay	501 ^c^	12.3 ^b^	1.14 ^b^	40.7 ^b^	438 ^b^	11.1 ^b^
	McMurray	501 ^b^	11.8 ^c^	1.01 ^d^	42.7 ^a^	494 ^a^	11.7 ^a^
	Anzac	507 ^a^	11.8 ^c^	1.04 ^c^	43.2 ^a^	487 ^a^	11.1 ^b^
*Vaccinium vitis-idaea*	Mildred	523 ^ab^	10.4 ^bc^	1.51 ^ab^	50.5 ^b^	347 ^b^	7.0 ^bc^
	JPH4	522 ^bc^	11.0 ^a^	1.42 ^bc^	47.3 ^c^	369 ^b^	7.8 ^a^
	McKay	525 ^a^	10.7 ^ab^	1.52 ^a^	49.0 ^c^	350 ^c^	7.1 ^c^
	McMurray	518 ^c^	9.7 ^d^	1.40 ^cd^	53.5 ^a^	371 ^ab^	7.1 ^bc^
	Anzac	524 ^a^	10.1 ^c^	1.41 ^d^	51.6 ^b^	370 ^a^	7.5 ^ab^
*Maianthemum trifolium*	Mildred	448 ^d^	28.8 ^b^	1.99 ^b^	15.5 ^a^	222 ^b^	14.7 ^a^
	JPH4	466 ^c^	31.9 ^a^	2.09 ^a^	14.9 ^c^	223^b^	16.2 ^a^
	McKay	471 ^bc^	31.8 ^ab^	2.14 ^ab^	14.9 ^bc^	225 ^b^	15.7 ^a^
	McMurray	476 ^a^	31.9 ^ab^	1.98 ^b^	14.9 ^bc^	244 ^a^	15.9 ^a^
	Anzac	470 ^b^	31.0 ^ab^	2.09 ^ab^	15.4 ^ab^	229 ^ab^	16.2 ^a^
*Rubus chamaemorus*	Mildred	473 ^c^	27.8 ^b^	2.51 ^a^	16.9 ^b^	191 ^d^	12.1 ^c^
	JPH4	486 ^b^	31.2 ^a^	2.02 ^b^	15.8 ^c^	240 ^c^	15.1 ^ab^
	McKay	488 ^b^	28.3 ^b^	1.79 ^c^	17.2 ^b^	270 ^b^	15.7 ^a^
	McMurray	491 ^a^	24.5 ^c^	1.61 ^d^	20.1 ^a^	305 ^a^	15.2 ^ab^
	Anzac	492 ^a^	29.0 ^a^	1.94 ^b^	17.1 ^bc^	255 ^c^	15.1 ^b^

Eight species (*E. mesomorpha*, *C. mitis*, *S. fuscum*, *S. capillifolium*, *P. mariana*, *V. oxycoccos*, *V. vitis-idaea*, *R. chamaemorus*) exhibited a pattern of exponentially decreasing N concentration and/or exponentially increasing C:N ratio with increasing distance from the Syncrude Mildred Lake and/or Suncor Base Plant upgrader stacks (Table S2). Similarly, seven species (*E. mesomorpha*, *C. mitis*, *S. fuscum*, *S. capillifolium*, *P. mariana*, *V. oxycoccos*, and *R. chamaemorus*) exhibited a pattern of exponentially decreasing S concentration and/or exponentially increasing C:S ratio with increasing distance from oil sands operations (Table S2).

For most species, tissue C concentrations increased with increasing distance from the Syncrude Mildred Lake and/or Suncor Base Plant upgrader stacks (Table 1), such that site differences in N or S tissue concentrations were amplified when expressed as C:N or C:S ratios (Table 1). Further, *R*^2^ values were generally higher for C:N and C:S ratio regressions as a function of distance from the upgrader stacks than for N or S concentration regressions (Table S2). For *S. fuscum*, *S. capillifolium*, *E. mesomorpha*, *C. mitis*, *V. oxycoccos*, and *P. mariana*, N:S ratios generally increased with increasing distance from oil sands operations (Table 1). Six of the plant/lichen species exhibited a significant decrease in C:N ratio over the course of the study at one or more sites; seven species exhibited a significant increase in C:S ratio over the course of the study at one or more sites (Table S3).

Six of the 10 species had tissue N concentrations that were positively correlated with growing season NH_4_^+^-N or NO_3_^−^-N deposition, and six species had tissue S concentrations that were positively correlated with growing season SO_4_^2−^-S deposition (Table S4). Seven of the 10 species had tissue C:N ratios that were negatively correlated with growing season NH_4_^+^-N, nine of the 10 species had tissue C:N ratios that were negatively correlated with growing season NO_3_^−^-N deposition, and six species had tissue C:S ratios that were positively correlated with growing season SO_4_^2−^-S deposition (Table S4).

### Power analysis

Power analyses revealed that the sampling intensity used throughout this study (5 replicates per species per collection date), at a *p* level of 0.10, produced power values ranging from 0.27 to 0.65 (Table 2). To achieve a power of at least 0.80 and a *p* level of 0.10, sampling would have to increase to between 6 and 20 replicates per species per collection date (Table 2).

### Influence of different temporal sampling intensities

Compared to results from our full data set (36 sampling dates over 8 years), using data from a single August collection in each of the 8 years led to less clear indications of site differences in plant/lichen tissue chemistry (Friedman’s test; Table S5), and when we used August data for 2010, 2013, and 2015 only, for many species, site differences in tissue chemistry that were clear in the full data set were not at all evident for many of the plant/lichen species (Table S6).

With regard to exponential regressions describing changes in N and S concentrations or C:N and C:S ratios from the Syncrude or Suncor upgrader stacks for each of the 10 plant/lichen species, using the full data set, of the 80 regressions, 50 were significant (*p* ≤ 0.0272; Table S2). Using annual August only data, five of these regressions were no longer significant, but in four instances, regressions that were not significant using the full data set became significant (Table S7). Using August data for 2010, 2013, and 2015 only, 13 of the regressions that were significant using the full data set were no longer significant, but in three instances, regressions that were not significant using the full data set became significant (Table S8).

When we used the full data set to examine changes in N and S concentrations or C:N and C:S ratios over the 8 years of the study, by species and site, 91 of the 200 correlations were significant (Table S3). Using the August only data for each year, 47 of the 91 were no longer significant, while 16 correlations that were not significant using the full data set became significant (Table S9). Using the August data 2010, 2013, and 2015 only, 57 of the 91 correlations that were significant using the full data set were no longer significant, while 14 correlations that were not significant using the full data set became significant (Table S10).

Of the 60 paired correlations between plant/lichen tissue N and S concentrations or C:N and C:S ratios with growing season NH_4_^+^-N, NO_3_^−^-N, or SO_4_^2−^-S deposition, 40 were significant (Table S4). Using the August only data, 20 of these became nonsignificant, while two that were not significant with the full data set became significant (Table S11). Using the August data for 2010, 2013, and 2015 only, 28 of the correlations that were significant using the full data set were no longer significant (Table S12).

## Discussion

### Plant/lichen tissue chemistry responses

Of the 10 plant/lichen species examined in this study, we anticipated that the two lichen species (*E. mesomorpha* and *C. mitis*), and the two *Sphagnum* species (*S. fuscum* and *S. capillifolium*), would have the highest biomonitoring potential. Because of the absence of roots and the uptake of nutrients, solutes, and gases across their entire thalli and capitula, lichens and Sphagna are quite dependent on the atmosphere for their supply of water and nutrients (Carter et al., 2017; Nash, 2008). Lichens have long been recognized as potentially useful indicators of atmospheric N and S pollution (e.g. Hawksworth & Rose, 1976; Pinho et al., 2017; Will-Wolf et al., 2017) as well as within the Alberta sands region (e.g., Graney et al., 2017; Landis et al., 2012, 2019). *Evernia mesomorpha* has been shown to be very sensitive to even short-term exposure to low doses of SO_2_, which reduces net CO_2_ assimilation and respiration rates as well as protein and lipid biosynthesis (e.g., Huebert et al., 1985; Malhotra & Khan, 1985). One of the earliest efforts to use lichens to assess the spatial patterns of elements, including S, released from oil sands activities (Addison & Puckett, 1980) showed that S concentrations in *E. mesomorpha* generally decreased (from > 3 mg g^−1^) with distance from the Suncor operations, which had been emitting about 150 metric tonnes of S da^−1^ since 1967. Further, *E. mesomorpha* became more luxuriant (semi-quantitative assessment) with distance from Suncor; lichen condition was related to S concentration. *E. mesomorpha* has long been regarded as an indicator species for NO_2_ exposure in the oil sands region (e.g. Addison & Puckett, 1980; Davies, 2012; Laxton et al., 2010), as well. Concentrations of N and S in *E. mesomorpha* tissues have been correlated with spatial patterns of atmospheric N and S deposition in the oil sands region (e.g. Davies, 2012; Wieder et al., 2016a).

We also observed that *E. mesomorpha* individuals were markedly smaller (1–2 cm^2^) at the JPH4 and Mildred sites than at sites farther from oil sands operations (4–6 cm^2^). Our work supports the use of *E. mesomorpha* as a biomonitor for changing atmospheric wet and dry N and S deposition regimes. Exposure of *E. mesomorpha* along N and S deposition gradients in the Alberta oil sands region is likely compromising the healthy status of this lichen.

Less research has focused on the terricolous lichen *C. mitis* as a potential biomonitor, although *C. mitis* has been regarded as an indicator species for SO_2_ across Canada (Thormann, 2006). In Finnish bogs, *C. mitis* S concentrations were positively correlated with atmospheric SO_4_^2−^-S deposition (Pakarinen, 1981). In a large-scale field fertilization experiment in Alberta, *C. mitis* N concentrations in apical tissues increased with increasing N addition (Bird et al., 2019). Concentrations of N and S in *C. mitis* tissues were positively correlated with spatial patterns of atmospheric N and S deposition in the Alberta oil sands region (Wieder et al., 2016a). However, tissue N and S responses to a gradient in N and S deposition were reported to be substantially stronger for *E. mesomorpha* than for *C. mitis* (Graney et al., 2017).

Based on our four criteria *E. mesomorpha* has potential as a biomonitor of changing N and S deposition regimes, and *C. mitis* may have some potential as a biomonitor for changing atmospheric deposition S, but not N regime (Table 3). Different responses of the lichens are consistent with a general finding that epiphytic lichens may be more responsive to increasing N and S inputs than terricolous lichens (cf. Graney et al., 2017; Watmough et al., 2019).

Because ombrotrophic bog *Sphagnum* mosses have a remarkable ability to retain atmospherically deposited N (Aldous, 2002; Hartsock et al., 2019; Jauhianen et al., 1998; Malmer et al., 1994), they have been used as indicators of atmospheric N deposition. Across three Swedish mire sites with gradient of wet and dry N deposition, as N deposition increased so did total N and amino acid N concentrations in *S. fuscum* (Wiederman et al., 2008). A survey of literature reporting N concentrations in *Sphagnum* species in European and North American peatlands indicated that as N deposition increased from 1 to 50 kg N ha^−1^ year^−1^, *Sphagnum* capitulum N concentrations increased from 5.5 to 13 mg g^−1^, following a sigmoid response (Lamers et al., 2000). At 16 bog sites across Europe, as N deposition increased from 0.8 to 20 kg N ha^−1^ year^−1^, N concentrations in *Sphagnum* capitula increased logarithmically from 5 to 13 mg g^−1^ (Bragazza et al., 2005). With field experimental N addition to Mer Bleue Bog, Ontario (up to 64 kg N ha^−1^ year^−1^), *S. capillifolium* capitulum N concentrations increased from 7 to 14 mg g^−1^ (Juutinen et al., 2015). In Alberta, the *Sphagnum* N concentration response to increasing N deposition is much less evident, in part because the N deposition gradient is less steep than in these other studies. *S. fuscum* N concentrations were not significantly different between Alberta bogs with N deposition ranging from 0.07 to 4.04 kg N ha^−1^ year^−1^ (Vitt et al., 2003). Across 20 bogs in the oil sands region, N concentrations in *S. fuscum* and *S. magellanicum* capitula were not significantly correlated with NH_4_^+^-N, NO_3_^−^-N, or DIN deposition (Wieder et al., 2016a). Field experimental addition of N to an Alberta bog at rates up to 25 kg N ha^−1^ year^−1^ led to small, but significant increases in *S. fuscum* N concentrations (Wieder et al., 2019). Biological N_2_-fixation, not atmospheric deposition, is the major source of new N to Alberta bogs (Vile et al., 2014) and is downregulated with increasing N deposition (Wieder et al., 2019). The dominance of N_2_-fixation over N deposition may buffer the responsiveness of *Sphagnum* N concentrations to increasing N deposition (Wieder et al., 2019).

Less research has focused on S concentrations is *Sphagnum* species as a function of S inputs. In Finnish bogs, *S. fuscum* S concentrations were positively correlated with atmospheric SO_4_^2−^-S deposition (Pakarinen, 1981), as was found for several *Sphagnum* species across Scandinavia (Malmer, 1988). Similarly, at 5 European bog sites, as throughfall S inputs increased, so did S concentrations in *Sphagnum* (Novák et al., 2001). At bogs in the Southern Pennines, UK, experimental exposure to HSO_3_^−^ and SO_4_^2−^ resulted in increased S concentrations in *S. recurvum* and *S. magellanicum* (Ferguson & Lee, 1980). For 20 bogs in the Alberta oil sands region, however, S concentrations in *S. fuscum*, but not in *S. capillifolium*, capitula were significantly correlated with SO_4_^2−^-S deposition (Wieder et al., 2016a). Based on our assessment criteria, *S. fuscum* has potential as a biomonitor of N deposition, while both species could serve as biomonitors of S deposition (Table 3).

**Table 2:** Power analysis to determine sample sizes required to detect 20% change in N or S concentration at *p* = 0.05 or 0.10 with a power ≥ 0.80

	Nitrogen (mg/g)		Sulfur (mg/g)	
	Sample size to detect 20% change, *p* = 0.10, Power ≥ 0.8	Power to detect 20% change, *p* = 0.10, *n* = 5	Sample size to detect 20% change, *p* = 0.10, Power ≥ 0.8	Power to detect 20% change, *p* = 0.10, *n* = 5
*Evernia mesomorpha*	8	0.54	12	0.40
*Cladonia mitis*	11	0.40	11	0.39
*Sphagnum fuscum*	9	0.46	15	0.33
*Sphagnum capillifolium*	10	0.44	20	0.27
*Picea mariana*	8	0.51	11	0.40
*Rhododendron groenlandicum*	11	0.39	11	0.40
*Vaccinium oxycoccos*	6	0.65	9	0.45
*Vaccinium vitis-idaea*	17	0.29	9	0.46
*Maianthemum trifolium*	10	0.44	12	0.37
*Rubus chamaemorus*	6	0.64	16	0.30
	C:N		C:S	
*Evernia mesomorpha*	8	0.51	13	0.36
*Cladonia mitis*	12	0.37	12	0.37
*Sphagnum fuscum*	9	0.46	9	0.46
*Sphagnum capillifolium*	12	0.39	15	0.33
*Picea mariana*	7	0.57	11	0.41
*Rhododendron groenlandicum*	8	0.51	9	0.49
*Vaccinium oxycoccos*	6	0.64	9	0.47
*Vaccinium vitis-idaea*	11	0.41	9	0.48
*Maianthemum trifolium*	15	0.33	12	0.37
*Rubus chamaemorus*	8	0.22	11	0.42

In contrast to lichens and mosses, there is less evidence that bog vascular plant species are effective biomonitors of N and S inputs. If the growth of a vascular plant species in a particular habitat is N-limited, increasing wet or dry N deposition could lead to a stimulation of growth without affecting plant/lichen tissue N concentrations. An increase in tissue N concentration generally indicates that N availability exceeds plant growth demands. However, it has been suggested that plants growing in low nutrient conditions, such as ombrotrophic bogs, may be less plastic in their growth response to a pulse of nutrient availability than plants growing in relatively high nutrient availability environments (Chapin et al., 1986). Thus, vascular plants in bogs may be likely to exhibit increased tissue N concentrations as N availability increases through increasing wet and/or dry deposition.

Field fertilization studies have shown that N addition can result in increased N concentrations in leaves of *R. groenlandicum* (Bubier et al., 2007; Juutinen et al., 2015), *Chamaedaphne calyculata* (Juutinen et al., 2015), and *V. oxycoccos* (Heijmans et al., 2001), with mixed results for *R. chamaemorus* (Nordbakken et al., 2003; van Heerwaarden et al., 2003). Previously, we found significant correlations between NH_4_^+^-N, NO_3_^−^-N, or DIN deposition and leaf/needle N concentrations for *R. groenlandicum* and *P. mariana*, but not for *V. oxycoccos* or *V. vitis-idaea*, as well as significant correlations between SO_4_^2−^-S deposition and leaf/needle S concentrations for *P. mariana*, but not for *R. groenlandicum, V. oxycoccos*, or *V. vitis-idaea* (Wieder et al., 2016a). Of the six vascular plant species, C:N ratios of *V. oxycoccos* leaves have the best potential for a biomonitor of changing N deposition regimes; C:S ratios of *V. oxycoccos* leaves and *P. mariana* needles have the best potential to serve as biomonitors of changing S deposition regimes (Table 3).

Ongoing monitoring of bogs should consider focusing on *E. mesomorpha*, *C. mitis*, *S. fuscum*, *S. capillifolium*, *P. mariana*, and *V. oxycoccos*. If a statistical power of 0.80 or greater is desired, sample sizes should be increased to 13, 12, 15, 20, 11, and 9, respectively (Table 2). Although *R. groenlandicum* appeared not to show strong potential as a biomonitor, in Alberta bogs, this species responds to increasing N deposition by increasing aboveground net primary production (Vitt et al., 2020; Wieder et al., 2019). If N deposition continues to increase to the point where *R. groenlandicum* growth is no longer N-limited, leaf tissue N concentrations may begin to increase, providing a signal of N saturation of the *R. groenlandicum* growth response. This could be the case for *V. vitis-idaea* as well, although we know of no net primary production measurements for this species in Alberta bogs. The perennial, deciduous forbs, *M. trifolium* and *R. chamaemorus*, have deep, aerenchymatous, non-mycorrhizal roots. Leaf N and S concentrations that are highest when leaves first appear and decrease throughout the growing season are likely the result of retranslocation of N and S from perennial roots along with uptake of N and S from deep within the peat profile. These two species have little value as biomonitors of changing N and S deposition regimes.

**Table 3:** Assessing species C:N and C:S ratios as indicators of a changing N and S deposition regime. For criterion 1, a single check indicates yes. For criterion 2, one check each for changing C:N or C:S ratios concentrations with distance from the Syncrude and Suncor upgrader stacks. For criterion 3, one check for each site that showed concentrations changing over time consistent with increasing N and decreasing S emissions. For criterion 4, one check each for plant/lichen C:N or C:S ratios correlating with growing season NH_4_^+^-N, NO_3_^−^-N, or SO_4_^2−^-S deposition

	Criterion 1	Criterion 2	Criterion 3	Criterion 4
Species	C:N or C:S ratios increase with distance from midpoint between Syncrude and Suncor stacks (Friedman’s Test)	C:N or C:S ratios increase with distance from Syncrude and Suncor stacks (exponential regression)	C:N or C:S change over time consistent with increasing N and decreasing S emissions (Kendall’s Tau)	C:N inversely correlated with growing season NH_4_^+^-N and/or NO_3_^−^-N deposition. C:S inversely correlated with growing season SO_4_^2−^-S deposition (Kendall’s Tau)
	Plant/lichen C:N			
*Evernia mesomorpha*	√	√√	√√	√√
*Cladonia mitis*	√	√√		
*Sphagnum fuscum*	√	√	√√√√	√√
*Sphagnum capillifolium*				√√
*Picea mariana*			√√√	√√
*Rhododendron groenlandicum*			√√	√√
*Vaccinium oxycoccos*	√	√√	√√√√√	√√
*Vaccinium vitis-idaea*	√	√√		√
*Maianthemum trifolium*			√√	√
*Rubus chamaemorus*		√√	√√√√	√√
	Plant/lichen C:S			
*Evernia mesomorpha*	√	√√	√√√√	√
*Cladonia mitis*	√	√√	√√√√	√
*Sphagnum fuscum*	√	√√	√√√	√
*Sphagnum capillifolium*	√	√√	√√√√√	√
*Picea mariana*	√	√√	√√√√√	
*Rhododendron groenlandicum*				
*Vaccinium oxycoccos*	√	√√	√√√√	√
*Vaccinium vitis-idaea*		√	√√	
*Maianthemum trifolium*				
*Rubus chamaemorus*		√√		√

As to temporal sampling intensity, comparing results from our full data set to two alternative sampling schemes, once per year in early August about half way through the growing season, or once every 2–3 years in early August, showed considerable diminishment of the ability to detect significant changes in plant/lichen tissue chemistry between sites (Table 1; Tables S5 and S6), with distance from the Syncrude and Suncor upgrader stacks (Tables S2, S7, and S8), over time (Tables S3, S9, and S10), and in response to growing season NH_4_^+^-N, NO_3_^−^-N, or SO_4_^2−^-S deposition (Tables S4, S11, and S12). We suggest that three sampling dates per year (June, July, August) are appropriate to best detect changes in plant/lichen tissue chemistry. This sampling frequency is especially critical in times when N and S emissions, and hence deposition to regional ecosystems, are changing (Fig. 1). If emissions and deposition were to stabilize at rather constant levels, a lower sampling intensity might be reasonable.

### Potential ecosystem-level consequences

While some bog plant/lichen species may be useful as monitors of changing N and S wet and dry deposition regimes, there may be ecosystem-level consequences. We have shown that experimental addition of N (as NH_4_NO_3_) to an Alberta bog resulted in increased tissue N concentrations for some plant species, but more critically led to an increase in cover of short-statured ericaceous shrubs, a change in *Sphagnum* species composition, and an overall decrease in *Sphagnum* abundance (Wieder et al., 2019), potentially compromising the net C sink function of bogs (Berendse et al., 2001; Bubier et al., 2007; Lamers et al., 2000; Limpens et al., 2011). Experimental augmentation of dry N deposition (20–56 kg NH_3_-N ha^−1^ year^−1^) at Whim Bog, Scotland, led to an almost complete loss of *Calluna vulgaris*, *S. capillifolium,* and *Cladonia portentosa,* while augmentation of wet N deposition (56 kg N ha^−1^ year^−1^ as NH_4_Cl) led to an increase in *Calluna* cover and decreased cover of *Sphagnum* and *Cladonia* (Sheppard et al., 2011). While these deposition levels are considerably higher than what is occurring in the oil sands region, they suggest the potential for substantial changes in vegetation as N deposition increases.

It is well recognized that although several lichen species may be useful as biomonitors of pollution, they also may be sensitive to SO_2_ and acid rain (Nash, 2008). Terricolous lichens may play a more important role in bogs that once realized. In the Hudson Bay lowlands, *Cladonia stellaris* and *Cladonia rangiferina* form thick mats that reduce *Sphagnum* cover and inhibit the growth of small shrubs (Harris et al., 2018). In Alberta, *C. mitis* also is abundant, so if a changing wet and dry N and S deposition regime begins to harm *C. mitis*, bog plant community structure could be altered. Further, given that both *E. mesomorpha* and *C. mitis* are an important food for woodland caribou (Edmonds & Bloomfield, 1984; Thomas et al., 1994; Thompson et al., 2012), any reduction in the abundance of this lichen would be undesirable.

Vascular plants can accumulate S, often storing it as SO_4_^2−^-S in vacuoles, and have other mechanisms of reducing S uptake, releasing S, or retranslocating S within a plant (Rennenberg, 1984). There is little evidence that SO_4_^2−^-S deposition has negative effects on bog vascular plants. However, SO_4_^2−^, SO_2_, and HSO_3_^−^ have been implicated as causally related to the decline of several *Sphagnum* species in the Great Britain (Ferguson & Lee, 1979, 1980; Ferguson et al., 1978). If SO_4_^2−^-S deposition remains higher than background levels in the oil sands region, anaerobic dissimilatory sulfate reduction may by stimulated, increasing anaerobic CO_2_ production while decreasing anaerobic CH_4_ production (Gauci et al., 2004; Vile et al., 2003). Relationships between SO_4_^2−^-S deposition, sulfate reduction, and the net fluxes of CO_2_ and CH_4_ have yet to be fully explored in the oil sands region.

## Conclusions

Emissions of NO_*x*_ and SO_2_ resulting from development of the oil sands resource in northern Alberta have substantially altered the atmospheric wet and dry N and S deposition regime. Peatlands, and bogs in particular, are naturally nutrient poor ecosystems. From an ecosystem perspective, bog structure and function are likely to be affected by changing N and S deposition. From a monitoring perspective, bog plant/lichen tissue chemistry may respond to changing N and S deposition and hence bog plant/lichen species could serve as biomonitors over both time and space. These two perspectives are complementary. Based on bog plant/lichen sampling from 5 sites from over 8 years period, we used three criteria to assess the biomonitor potential of plant/lichen species: (1) whether each species showed changes in tissue chemistry with increasing distance from the Syncrude and Suncor upgrader stacks (the two largest point sources of N and S emissions); (2) whether tissue chemistry changed over the 8 year period in ways that were consistent with increasing N and decreasing S emissions from oil sands facilities; and (3) whether tissue chemistry was correlated with growing season wet deposition of NH_4_^+^-N, NO_3_^−^-N, or SO_4_^2−^-S. Based on these criteria, the best biomonitors of a changing N deposition regime were *Evernia mesomorpha*, *Sphagnum fuscum*, and *Vaccinium oxycoccos*. The best biomonitors of a changing S deposition regime were *Evernia mesomorpha*, *Cladonia mitis*, *Sphagnum fuscum*, *Sphagnum capillifolium*, *Vaccinium oxycoccos*, and *Picea mariana*. Further, we show that as sampling frequency decreases from multiple plant/lichen collections within years over 8 years, to once per year over 8 years, to once per year every 2–3 years, the reliability of the plant/lichen tissue chemistry as biomonitors of changing N and S deposition decreases. Throughout this study, we collected 5 replicate samples of each species on each sampling date. Power analysis indicates that to be able to detect a 20% change in plant/lichen N or S tissue concentrations between two sites or sampling dates at *p* = 0.10, sample sizes should be increased to between 6 and 20, depending on the species.

## Supplementary Information

Below is the link to the electronic supplementary material.

Supplementary file1 (PDF 512 KB)

## Data Availability

Plant/lichen tissue C, N, and S data are available through the Environmental Data Initiative (10.6073/pasta/b84f468bccfa3651c6d8fed6833cf085).
