# Supplementary material for: Bog plant/lichen tissue nitrogen and sulfur concentrations as indicators of emissions from oil sands development in Alberta, Canada
Source: Environ Monit Assess. 2021 Mar 23;193(4):208. doi: 10.1007/s10661-021-08929-y (PMC7987692; doi:10.1007/s10661-021-08929-y)
Supplement: Supplementary file 1 — Supplementary file1 (PDF 512 KB) [file 10661_2021_8929_MOESM1_ESM.pdf]

**Supplemental Material**

Authors: R. Kelman Wieder, Melanie A. Vile, Kimberli D. Scott, Cara M. Albright, James C. Quinn, D.H. Vitt

Title: Bog plant/lichen tissue nitrogen and sulfur concentrations as indicators of emissions from oil sands development in Alberta, Canada

Journal: *Environmental Monitoring and Assessment*

Table S1. Precision (standard deviation of 12 replicate analyses) and accuracy (comparison of measured values with certified concentrations) for N and S analyses. Coefficients of variation for N and S measurements averaged 1.0 and 3.1 %, respectively. On average, mean measured N and S concentrations were within 2.2 and 5.4 % of certified values, respectively.

| | Reference material | | |
|---|---|---|---|
| | Apple Leaves (NIST 1515) | Tomato Leaves (NIST 1573a) | Orchard Leaves (LECO 1027) |
| Nitrogen (mg $g^{-1}$) | | | |
| Mean ± standard deviation ($n$ = 12) | 23.4 ± 0.2 | 31.5 ± 0.4 | 19.7 ± 0.2 |
| Certified values | 22.5 ± 1.9 | 30.3 ± 1.5 | 20.0 ± 0.3 |
| | Coal Fly Ash (NIST 1633b) | Alfalfa (LECO 1021) | Barley Flour (LECO 1009) |
| Sulfur (mg $g^{-1}$) | | | |
| Mean ± standard deviation ($n$ = 12) | 1.96 ± 0.04 | 2.19 ± 0.11 | 1.38 ± 0.03 |
| Certified values | 2.07 ± 0.01 | 2.42 ± 0.11 | 1.38 ± 0.06 |

Table S2. Regression parameters describing exponential decrease in tissue N and S concentrations and exponential increase in C:N or C:S ratios with distance from the Syncrude Mildred Lake and Suncor Base Plant upgrader stacks. Blank cells indicate that either the regression did not converge or was nonsignificant. N or S concentration = $A + Be^{-(C \times distance)}$; C:N or C:S ratios = $A + B(1-e^{-(C \times distance)})$. Data from the spring collections for which N concentrations peaked (Fig. 1) were excluded from the analysis.

| | | Tissue N regressions | | Tissue S regressions | | C:N regressions | | C:S regressions | |
|---|---|---|---|---|---|---|---|---|---|
| Species | Parameter | Syncrude; Mildred Lake | Suncor; Base Plant | Syncrude; Mildred Lake | Suncor; Base Plant | Syncrude; Mildred Lake | Suncor; Base Plant | Syncrude; Mildred Lake | Suncor; Base Plant |
| *Evernia mesomorpha* | A | 8.3 | 8.5 | 1.1 | 1.0 | 5.8 | 30.0 | 75.7 | 220.7 |
| | B | 13.8 | 8.0 | 1.9 | 0.8 | 49.4 | 25.5 | 361.0 | 266.2 |
| | C | 0.14 | 0.15 | 0.10 | 0.06 | 0.09 | 0.07 | 0.06 | 0.03 |
| | $R^2$ | 0.31 | 0.40 | 0.40 | 0.45 | 0.41 | 0.43 | 0.45 | 0.47 |
| | *p* | <0.0001 | <0.0001 | <0.0001 | <0.0001 | <0.0001 | <0.0001 | <0.0001 | <0.0001 |
| *Cladonia mitis* | A | 5.1 | 5.1 | 0.6 | 0.5 | 23.2 | 61.0 | 251.7 | 484.9 |
| | B | 4.4 | 3.3 | 0.6 | 0.3 | 66.9 | 29.3 | 590.5 | 404.0 |
| | C | 0.13 | 0.16 | 0.08 | 0.05 | 0.12 | 0.09 | 0.06 | 0.03 |
| | $R^2$ | 0.09 | 0.12 | 0.26 | 0.28 | 0.10 | 0.12 | 0.31 | 0.30 |
| | *p* | <0.0001 | <0.0001 | <0.0001 | <0.0001 | <0.0001 | <0.0001 | <0.0001 | <0.0001 |
| *Sphagnum fuscum* | A | 12.1 | 11.7 | 1.1 | 1.1 | 26.3 | 32.9 | 25.9 | 180.5 |
| | B | 1.6 | 1.4 | 1.8 | 1.3 | 12.5 | 7.4 | 393.5 | 280.2 |
| | C | 0.05 | 0.02 | 0.07 | 0.06 | 0.08 | 0.03 | 0.06 | 0.03 |
| | $R^2$ | 0.03 | 0.02 | 0.36 | 0.41 | 0.09 | 0.09 | 0.51 | 0.51 |
| | *p* | <0.0001 | 0.0004 | <0.0001 | <0.0001 | <0.0001 | <0.0001 | <0.0001 | <0.0001 |
| *Sphagnum capillifolium* | A | | | 1.1 | 1.2 | | 34.6 | 56.6 | 225.0 |
| | B | | | 1.4 | 1.2 | | 3.0 | 367.1 | 206.7 |
| | C | | | 0.08 | 0.11 | | 0.04 | 0.08 | 0.05 |
| | $R^2$ | | | 0.21 | 0.25 | | 0.01 | 0.29 | 0.29 |
| | *p* | | | <0.0001 | <0.0001 | | 0.0141 | <0.0001 | <0.0001 |
| *Picea mariana* | A | | | | 0.8 | | 45.9 | | 382.4 |
| | B | | | | 0.7 | | 21.4 | | 287.1 |

| | | | | | | | | | |
|---|---|---|---|---|---|---|---|---|---|
| | C | | | | 0.13 | | 0.16 | | 0.07 |
| | $R^2$ | | | | 0.19 | | 0.03 | | 0.19 |
| | *p* | | | | <0.0001 | | <0.0001 | | <0.0001 |
| *Vaccinium oxycoccos* | A | 11.6 | 8.4 | 1.0 | 1.0 | 32.8 | 37.7 | 287.1 | 374.1 |
| | B | 2.9 | 4.9 | 0.6 | 0.3 | 11.8 | 15.2 | 213.9 | 136.7 |
| | C | 0.05 | 0.01 | 0.08 | 0.05 | 0.05 | 0.01 | 0.07 | 0.04 |
| | $R^2$ | 0.09 | 0.08 | 0.15 | 0.16 | 0.12 | 0.11 | 0.17 | 0.16 |
| | *p* | <0.0001 | <0.0001 | <0.0001 | <0.0001 | <0.0001 | <0.0001 | <0.0001 | <0.0001 |
| *Vaccinium vitis-idaea* | A | 10.0 | 9.1 | | | 44.3 | 48.5 | | |
| | B | 2.0 | 2.0 | | | 8.5 | 7.3 | | |
| | C | 0.05 | 0.01 | | | 0.05 | 0.01 | | |
| | $R^2$ | 0.05 | 0.03 | | | 0.05 | 0.03 | | |
| | *p* | <0.0001 | 0.0002 | | | <0.0001 | <0.0001 | | |
| *Rubus chamaemorus* | A | 26.5 | 26.8 | 1.8 | 1.8 | 8.4 | 15.5 | | |
| | B | 13.5 | 3.7 | 4.1 | 14.6 | 11.5 | 4.1 | | |
| | C | 0.11 | 0.05 | 0.16 | 0.35 | 0.10 | 0.06 | | |
| | $R^2$ | 0.03 | 0.01 | 0.10 | 0.21 | 0.05 | 0.03 | | |
| | *p* | 0.0002 | 0.0272 | <0.0001 | <0.0001 | <0.0001 | <0.0001 | | |

Table S3. Correlations (Kendall's Tau) between tissue N or S concentrations, C:N ratios, and C:S ratios and time (years). Significant correlations (one-way tests for increasing N, decreasing C:N, decreasing S, increasing C:S) are highlighted in bold italic font.

| Species | Element or Ratio | | Site: Mildred | JPH4 | McKay | McMurray | Anzac |
|---|---|---|---|---|---|---|---|
| *Cladonia mitis* | N | *τ* | 0.0582 | -0.1673 | -0.0599 | -0.2072 | 0.0429 |
| | | *p* | 0.4121 | 0.0073 | 0.3024 | 0.0003 | 0.4601 |
| | C:N | *τ* | -0.1118 | 0.1766 | 0.0422 | 0.1758 | -0.0311 |
| | | *p* | 0.1144 | 0.0046 | 0.4671 | 0.0023 | 0.5920 |
| | S | *τ* | ***-0.3389*** | 0.0821 | ***-0.3391*** | ***-0.3677*** | ***-0.3450*** |
| | | *p* | ***<0.0001*** | 0.1907 | ***<0.0001*** | ***<0.0001*** | ***<0.0001*** |
| | C:S | *τ* | ***0.2853*** | -0.0341 | ***0.3018*** | ***0.3539*** | ***0.3551*** |
| | | *p* | ***<0.0001*** | 0.5852 | ***<0.0001*** | ***<0.0001*** | ***<0.0001*** |
| *Evernia mesomorpha* | N | *τ* | 0.0411 | 0.0646 | -0.0449 | 0.0549 | -0.0970 |
| | | *p* | 0.5607 | 0.3007 | 0.4357 | 0.3442 | 0.0923 |
| | C:N | *τ* | -0.0955 | ***-0.1957*** | 0.0248 | ***-0.1026*** | 0.0751 |
| | | *p* | 0.1758 | ***0.0017*** | 0.6667 | ***0.0765*** | 0.1920 |
| | S | *τ* | ***-0.5064*** | -0.0357 | ***-0.3390*** | ***-0.3362*** | ***-0.3375*** |
| | | *p* | ***<0.0001*** | 0.5679 | ***<0.0001*** | ***<0.0001*** | ***<0.0001*** |
| | C:S | *τ* | ***0.4584*** | 0.0708 | ***0.3147*** | ***0.2978*** | ***0.3068*** |
| | | *p* | ***<0.0001*** | 0.2558 | ***<0.0001*** | ***<0.0001*** | ***<0.0001*** |
| *Sphagnum capillifolium* | N | *τ* | -0.0655 | -0.2835 | -0.2285 | -0.2051 | -0.0648 |
| | | *p* | 0.3707 | <0.0001 | 0.0002 | 0.0008 | 0.2724 |
| | C:N | *τ* | -0.0024 | 0.1415 | 0.1282 | 0.0653 | -0.0582 |
| | | *p* | 0.9732 | 0.0237 | 0.0340 | 0.2844 | 0.3309 |
| | S | *τ* | ***-0.1404*** | ***-0.1585*** | ***-0.3061*** | ***-0.3549*** | ***-0.3088*** |
| | | *p* | ***0.0478*** | ***0.0111*** | ***<0.0001*** | ***<0.0001*** | ***<0.0001*** |
| | C:S | *τ* | 0.0789 | 0.0426 | ***0.1821*** | ***0.2485*** | ***0.2094*** |
| | | *p* | 0.2728 | 0.4953 | ***0.0026*** | ***<0.0001*** | ***0.0005*** |
| *Sphagnum fuscum* | N | *τ* | ***0.3624*** | 0.0576 | ***0.1794*** | ***0.1242*** | ***0.3194*** |
| | | *p* | ***<0.0001*** | 0.3446 | ***0.0021*** | ***0.0312*** | ***<0.0001*** |
| | C:N | *τ* | ***-0.3887*** | -0.0965 | ***-0.1630*** | ***-0.1072*** | ***-0.3302*** |
| | | *p* | ***<0.0001*** | 0.1129 | ***0.0052*** | ***0.0625*** | ***<0.0001*** |
| | S | *τ* | -0.0398 | 0.0222 | ***-0.2661*** | ***-0.2903*** | ***-0.2580*** |
| | | *p* | 0.5771 | 0.7201 | ***<0.0001*** | ***<0.0001*** | ***<0.0001*** |
| | C:S | *τ* | 0.0302 | -0.0216 | ***0.2570*** | ***0.2591*** | ***0.2146*** |

| | | | | | | | |
|---|---|---|---|---|---|---|---|
| | | *p* | 0.6747 | 0.7273 | ***<0.0001*** | ***<0.0001*** | ***0.0002*** |
| *Picea mariana* | N | *τ* | ***0.2533*** | 0.0233 | ***0.1353*** | -0.0279 | 0.0373 |
| | | *p* | ***0.0004*** | 0.7095 | ***0.0196*** | 0.6305 | 0.5199 |
| | C:N | *τ* | ***-0.2482*** | 0.0016 | ***-0.1069*** | 0.0504 | -0.0025 |
| | | *p* | ***0.0005*** | 0.9796 | ***0.0652*** | 0.3841 | 0.9654 |
| | S | *τ* | ***-0.2589*** | ***-0.1618*** | ***-0.2493*** | ***-0.3114*** | ***-0.4013*** |
| | | *p* | ***0.0003*** | ***0.0102*** | ***<0.0001*** | ***<0.0001*** | ***<0.0001*** |
| | C:S | *τ* | ***0.2838*** | ***0.1748*** | ***0.2591*** | ***0.3188*** | ***0.4077*** |
| | | *p* | ***<0.0001*** | ***0.0052*** | ***<0.0001*** | ***<0.0001*** | ***<0.0001*** |
| *Rhododendron groenlandicum* | N | *τ* | ***0.2460*** | 0.0208 | -0.0107 | 0.0602 | ***0.1546*** |
| | | *p* | ***0.0005*** | 0.7374 | 0.8525 | 0.2945 | ***0.0071*** |
| | C:N | *τ* | ***-0.2715*** | -0.0477 | -0.0446 | ***-0.1016*** | ***-0.2022*** |
| | | *p* | ***0.0001*** | 0.4419 | 0.4374 | ***0.0767*** | ***0.0004*** |
| | S | *τ* | -0.1189 | 0.1840 | 0.0409 | -0.0173 | 0.0258 |
| | | *p* | 0.1039 | 0.0031 | 0.4787 | 0.7630 | 0.6538 |
| | C:S | *τ* | 0.1144 | -0.2334 | -0.0798 | 0.0316 | -0.0489 |
| | | *p* | 0.1169 | 0.0002 | 0.1655 | 0.5813 | 0.3945 |
| *Vaccinium oxycoccos* | N | *τ* | ***0.1983*** | ***0.2114*** | ***0.3434*** | ***0.2120*** | ***0.3485*** |
| | | *p* | ***0.0176*** | ***0.0007*** | ***<0.0001*** | ***0.0002*** | ***<0.0001*** |
| | C:N | *τ* | -0.1136 | ***-0.2181*** | ***-0.3284*** | ***-0.1955*** | ***-0.3687*** |
| | | *p* | 0.1089 | ***0.0004*** | ***<0.0001*** | ***0.0007*** | ***<0.0001*** |
| | S | *τ* | ***-0.3264*** | ***-0.2365*** | ***-0.1316*** | -0.0739 | ***-0.1758*** |
| | | *p* | ***<0.0001*** | ***0.0002*** | ***0.0236*** | 0.2002 | ***0.0032*** |
| | C:S | *τ* | ***0.3536*** | ***0.2537*** | ***0.1907*** | ***0.1037*** | ***0.1600*** |
| | | *p* | ***<0.0001*** | ***<0.0001*** | ***0.0010*** | ***0.0716*** | ***0.0065*** |
| *Vaccinium vitis-idaea* | N | *τ* | -0.1552 | -0.1243 | -0.0735 | -0.1000 | -0.0135 |
| | | *p* | 0.0278 | 0.0464 | 0.2024 | 0.0867 | 0.8141 |
| | C:N | *τ* | 0.0995 | 0.0898 | 0.0155 | 0.0313 | -0.0054 |
| | | *p* | 0.1579 | 0.1498 | 0.7873 | 0.5916 | 0.9258 |
| | S | *τ* | ***-0.2500*** | ***-0.1213*** | ***-0.1441*** | -0.0779 | ***-0.2579*** |
| | | *p* | ***0.0004*** | ***0.0520*** | ***0.0485*** | 0.1854 | ***<0.0001*** |
| | C:S | *τ* | ***0.1696*** | 0.0871 | 0.0745 | 0.0251 | ***0.2261*** |
| | | *p* | ***0.0161*** | 0.1623 | 0.1974 | 0.6700 | ***<0.0001*** |
| *Maianthemum trifolium* | N | *τ* | ***0.3203*** | 0.2605 | 0.0426 | ***0.2552*** | 0.2070 |
| | | *p* | ***0.0112*** | 0.1121 | 0.5141 | ***0.0002*** | 0.1541 |
| | C:N | *τ* | ***-0.3309*** | -0.2608 | -0.0648 | ***-0.2757*** | -0.2200 |
| | | *p* | ***0.0087*** | 0.1121 | 0.3205 | ***<0.0001*** | 0.1300 |

| | | | | | | | |
|---|---|---|---|---|---|---|---|
| | S | $\tau$ | 0.1281 | -0.2415 | -0.1065 | 0.1367 | -0.0631 |
| | | *p* | 0.3049 | 0.1407 | 0.1025 | 0.0445 | 0.6710 |
| | C:S | $\tau$ | 0.1933 | 0.2415 | 0.0810 | -0.1521 | 0.0083 |
| | | *p* | 0.1316 | 0.1407 | 0.2167 | 0.0252 | 0.9574 |
| *Rubus chamaemorus* | N | $\tau$ | 0.1655 | ***0.2042*** | ***0.3489*** | ***0.2440*** | ***0.2176*** |
| | | *p* | 0.1437 | ***0.0036*** | ***<0.0001*** | ***0.0001*** | ***0.0012*** |
| | C:N | $\tau$ | -0.1608 | ***-0.2080*** | ***-0.3712*** | ***-0.2390*** | ***-0.2297*** |
| | | *p* | 0.1554 | ***0.0030*** | ***<0.0001*** | ***<0.0001*** | ***0.0006*** |
| | S | $\tau$ | 0.1002 | 0.0622 | 0.1468 | 0.2661 | 0.1970 |
| | | *p* | 0.3663 | 0.3731 | 0.0212 | <0.0001 | 0.0037 |
| | C:S | $\tau$ | -0.1092 | -0.0652 | -0.1648 | -0.2613 | -0.2129 |
| | | *p* | 0.3349 | 0.3520 | 0.0096 | <0.0001 | 0.0017 |

Table S4. Kendall's correlation coefficients (***τ***) and *p* values for tissue N or S concentrations, C:N and C:S ratios with growing season $NH_4^+$-N, $NO_3^-$-N, or $SO_4^{2-}$-S deposition (from Wieder et al. 2016b). Significant correlations (one-sided test for positive correlation) are indicated in bold italic font.

| Species | | N concentration with growing season $NH_4^+$-N deposition | N concentration with growing season $NO_3^-$-N deposition | S concentration with growing season $SO_4^{2-}$-S deposition | C:N with growing season $NH_4^+$-N deposition | C:N with growing season $NO_3^-$-N deposition | C:S with growing season $SO_4^{2-}$-S deposition |
|---|---|---|---|---|---|---|---|
| *Cladonia mitis* | ***τ*** | 0.0036 | -0.0371 | ***0.1448*** | 0.0148 | 0.0254 | ***-0.1460*** |
| | *p* | 0.8875 | 0.1481 | ***<0.0001*** | 0.5650 | 0.3216 | ***<0.0001*** |
| *Evernia mesomorpha* | ***τ*** | ***0.1220*** | ***0.2701*** | ***0.1524*** | ***-0.1166*** | ***-0.2474*** | ***-0.2040*** |
| | *p* | ***<0.0001*** | ***<0.0001*** | ***<0.0001*** | ***<0.0001*** | ***<0.0001*** | ***<0.0001*** |
| *Sphagnum capillifolium* | ***τ*** | 0.0105 | -0.0164 | ***0.1613*** | ***-0.0700*** | ***-0.0653*** | ***-0.2131*** |
| | *p* | 0.6915 | 0.5345 | ***<0.0001*** | ***0.0081*** | ***0.0134*** | ***<0.0001*** |
| *Sphagnum fuscum* | ***τ*** | ***0.1248*** | ***0.1551*** | ***0.2361*** | ***-0.1294*** | ***-0.2184*** | ***-0.2502*** |
| | *p* | ***<0.0001*** | ***<0.0001*** | ***<0.0001*** | ***<0.0001*** | ***<0.0001*** | ***<0.0001*** |
| *Picea mariana* | ***τ*** | ***0.2144*** | ***0.1062*** | 0.0362 | ***-0.1912*** | ***-0.1025*** | -0.0261 |
| | *p* | ***<0.0001*** | ***<0.0001*** | 0.1579 | ***<0.0001*** | ***<0.0001*** | 0.3086 |
| *Rhododendron groenlandicum* | ***τ*** | ***0.1023*** | 0.0503 | -0.0039 | ***-0.1066*** | ***-0.0494*** | -0.0146 |
| | *p* | ***<0.0001*** | 0.1139 | 0.8794 | ***<0.0001*** | ***0.0523*** | 0.5685 |
| *Vaccinium oxycoccos* | ***τ*** | ***0.1036*** | ***0.2378*** | ***0.0855*** | ***-0.0753*** | ***-0.2558*** | ***-0.0641*** |
| | *p* | ***<0.0001*** | ***<0.0001*** | ***0.0009*** | ***0.0034*** | ***<0.0001*** | ***0.0126*** |
| *Vaccinium vitis-idaea* | ***τ*** | 0.0091 | 0.0413 | -0.0165 | -0.0204 | ***-0.0871*** | -0.0047 |
| | *p* | 0.7225 | 0.1068 | 0.5207 | 0.4260 | ***0.0007*** | 0.8530 |
| *Maianthemum trifolium* | ***τ*** | 0.0090 | ***0.1357*** | 0.0042 | -0.0119 | ***-0.1392*** | -0.0079 |
| | *p* | 0.8160 | ***0.0005*** | 0.9125 | 0.7582 | ***0.0003*** | 0.8395 |
| *Rubus chamaemorus* | ***τ*** | ***0.0591*** | ***0.1740*** | ***0.0708*** | ***-0.0640*** | ***-0.1888*** | ***-0.0713*** |
| | *p* | ***0.0480*** | ***<0.0001*** | ***0.0177*** | ***0.0322*** | ***<0.0001*** | ***0.0172*** |

Table S5. Median tissue concentrations of C, N, and S, and C:N, C:S, and N:S ratios for the August sampling dates only for each of the eight years. For each parameter and each species, medians with the same letter superscript do not differ significantly ($p < 0.05$) per Friedman's test (sampling date as the blocked factor); *a posteriori* comparisons made using Tukey's Honestly Significant Difference Test ($\alpha = 0.05$) (Pereira et al. 2015).

| Species | Site | C (mg/g) | N (mg/g) | S (mg/g) | C:N | C:S | N:S |
|---|---|---|---|---|---|---|---|
| *Evernia mesomorpha* | Mildred | 420 $^{d}$ | 11.3 $^{a}$ | 1.62 $^{a}$ | 36.8 $^{d}$ | 255 $^{d}$ | 6.9 $^{b}$ |
| | JPH4 | 423 $^{c}$ | 9.7 $^{b}$ | 1.41 $^{b}$ | 44.3 $^{c}$ | 313 $^{c}$ | 6.9 $^{b}$ |
| | McKay | 424 $^{c}$ | 8.8 $^{c}$ | 1.27 $^{c}$ | 47.9 $^{b}$ | 334 $^{b}$ | 7.3 $^{b}$ |
| | McMurray | 454 $^{a}$ | 8.0 $^{c}$ | 1.11 $^{d}$ | 56.4 $^{a}$ | 408 $^{a}$ | 7.8 $^{a}$ |
| | Anzac | 453 $^{a}$ | 8.7 $^{cd}$ | 1.01 $^{d}$ | 53.0 $^{a}$ | 454 $^{a}$ | 8.2 $^{a}$ |
| *Cladonia mitis* | Mildred | 431 $^{b}$ | 5.4 $^{a}$ | 0.72 $^{a}$ | 79.3 $^{b}$ | 582 $^{c}$ | 7.4 $^{b}$ |
| | JPH4 | 433 $^{b}$ | 4.9 $^{ab}$ | 0.63 $^{b}$ | 88.7 $^{b}$ | 687 $^{bc}$ | 8.3 $^{b}$ |
| | McKay | 440 $^{b}$ | 4.7 $^{b}$ | 0.60 $^{b}$ | 90.3 $^{ab}$ | 690 $^{b}$ | 8.0 $^{b}$ |
| | McMurray | 441 $^{a}$ | 4.7 $^{b}$ | 0.47 $^{c}$ | 95.4 $^{a}$ | 935 $^{a}$ | 9.6 $^{a}$ |
| | Anzac | 448 $^{a}$ | 4.9 $^{ab}$ | 0.48 $^{c}$ | 90.4 $^{ab}$ | 923 $^{a}$ | 9.6 $^{a}$ |
| *Sphagnum fuscum* | Mildred | 437 $^{c}$ | 13.3 $^{a}$ | 1.79 $^{a}$ | 32.2 $^{b}$ | 245 $^{c}$ | 7.4 $^{c}$ |
| | JPH4 | 447 $^{b}$ | 14.0 $^{a}$ | 1.50 $^{a}$ | 31.9 $^{b}$ | 296 $^{c}$ | 9.4 $^{bc}$ |
| | McKay | 456 $^{b}$ | 13.3 $^{a}$ | 1.37 $^{b}$ | 34.8 $^{ab}$ | 329 $^{b}$ | 9.9 $^{b}$ |
| | McMurray | 457 $^{b}$ | 12.8 $^{a}$ | 1.02 $^{c}$ | 35.6 $^{ab}$ | 449 $^{a}$ | 12.3 $^{a}$ |
| | Anzac | 464 $^{a}$ | 12.4 $^{a}$ | 1.07 $^{c}$ | 37.7 $^{a}$ | 425 $^{a}$ | 12.0 $^{a}$ |
| *Sphagnum capillifolum* | Mildred | 453 $^{b}$ | 13.0 $^{b}$ | 1.55 $^{a}$ | 34.3 $^{abc}$ | 302 $^{c}$ | 8.6 $^{b}$ |
| | JPH4 | 444 $^{b}$ | 13.1 $^{ab}$ | 1.32 $^{a}$ | 33.9 $^{bc}$ | 328 $^{bc}$ | 10.1 $^{b}$ |
| | McKay | 459 $^{a}$ | 13.5 $^{a}$ | 1.24 $^{a}$ | 33.1 $^{c}$ | 374 $^{b}$ | 11.4 $^{a}$ |
| | McMurray | 453 $^{a}$ | 12.5 $^{b}$ | 1.05 $^{b}$ | 36.9 $^{a}$ | 436 $^{a}$ | 11.2 $^{a}$ |
| | Anzac | 460 $^{a}$ | 13.2 $^{b}$ | 1.05 $^{b}$ | 35.2 $^{ab}$ | 449 $^{a}$ | 12.2 $^{a}$ |
| *Picea mariana* | Mildred | 514 $^{a}$ | 8.1 $^{a}$ | 0.95 $^{a}$ | 63.5 $^{c}$ | 542 $^{c}$ | 8.3 $^{c}$ |
| | JPH4 | 510 $^{c}$ | 7.8 $^{a}$ | 0.91 $^{a}$ | 64.8 $^{c}$ | 565 $^{c}$ | 8.9 $^{c}$ |
| | McKay | 510 $^{bc}$ | 6.9 $^{c}$ | 0.78 $^{b}$ | 73.3 $^{a}$ | 655 $^{b}$ | 8.9 $^{bc}$ |
| | McMurray | 513 $^{ab}$ | 7.0 $^{ab}$ | 0.70 $^{b}$ | 72.5 $^{bc}$ | 730 $^{a}$ | 10.4 $^{a}$ |
| | Anzac | 513 $^{ab}$ | 7.0 $^{bc}$ | 0.73 $^{b}$ | 71.8 $^{ab}$ | 705 $^{ab}$ | 9.7 $^{ab}$ |
| *Rhododendron groenlandicum* | Mildred | 546 $^{b}$ | 15.8 $^{ab}$ | 1.22 $^{a}$ | 33.5 $^{ab}$ | 445 $^{ab}$ | 13.3 $^{ab}$ |
| | JPH4 | 550 $^{a}$ | 15.6 $^{ab}$ | 1.25 $^{a}$ | 35.0 $^{a}$ | 444 $^{b}$ | 13.8 $^{b}$ |
| | McKay | 547 $^{ab}$ | 15.5 $^{ab}$ | 1.22 $^{a}$ | 34.6 $^{ab}$ | 450 $^{b}$ | 13.5 $^{b}$ |
| | McMurray | 550 $^{ab}$ | 15.4 $^{b}$ | 1.06 $^{b}$ | 34.9 $^{a}$ | 511 $^{a}$ | 15.1 $^{a}$ |

| | | | | | | | |
|---|---|---|---|---|---|---|---|
| | Anzac | 550 $^{a}$ | 16.4 $^{a}$ | 1.24 $^{a}$ | 33.1 $^{b}$ | 451 $^{b}$ | 13.6 $^{ab}$ |
| *Vaccinium oxycoccos* | Mildred | 503 $^{bc}$ | 13.2 $^{a}$ | 1.19 $^{a}$ | 38.1 $^{b}$ | 424 $^{b}$ | 10.3 $^{a}$ |
| | JPH4 | 504 $^{c}$ | 14.1 $^{a}$ | 1.24 $^{a}$ | 35.3 $^{b}$ | 404 $^{b}$ | 11.3 $^{a}$ |
| | McKay | 504 $^{c}$ | 13.6 $^{a}$ | 1.20 $^{a}$ | 37.0 $^{b}$ | 418 $^{b}$ | 11.3 $^{a}$ |
| | McMurray | 504 $^{ab}$ | 12.8 $^{b}$ | 1.09 $^{b}$ | 39.5 $^{a}$ | 466 $^{a}$ | 11.7 $^{a}$ |
| | Anzac | 508 $^{a}$ | 12.8 $^{b}$ | 1.05 $^{b}$ | 40.8 $^{a}$ | 485 $^{a}$ | 12.0 $^{a}$ |
| *Vaccinium vitis-idaea* | Mildred | 527 $^{b}$ | 10.3 $^{abc}$ | 1.40 $^{ab}$ | 51.3 $^{abc}$ | 367 $^{a}$ | 7.3 $^{ab}$ |
| | JPH4 | 523 $^{ab}$ | 10.9 $^{a}$ | 1.34 $^{b}$ | 47.3 $^{c}$ | 387 $^{a}$ | 8.3 $^{a}$ |
| | McKay | 525 $^{a}$ | 10.6 $^{ab}$ | 1.47 $^{a}$ | 49.3 $^{bc}$ | 361 $^{b}$ | 7.2 $^{b}$ |
| | McMurray | 521 $^{b}$ | 9.7 $^{c}$ | 1.36 $^{ab}$ | 53.4 $^{a}$ | 382 $^{ab}$ | 7.4 $^{b}$ |
| | Anzac | 526 $^{ab}$ | 9.9 $^{bc}$ | 1.33 $^{b}$ | 52.8 $^{ab}$ | 391 $^{a}$ | 7.7 $^{ab}$ |
| *Maianthemum trifolia* | Mildred | 447 $^{c}$ | 25.7 $^{a}$ | 1.85 $^{a}$ | 17.2 $^{ab}$ | 237 $^{a}$ | 15.0 $^{a}$ |
| | JPH4 | 470 $^{bc}$ | 27.2 $^{a}$ | 1.78 $^{a}$ | 17.4 $^{ab}$ | 265 $^{a}$ | 16.4 $^{a}$ |
| | McKay | 474 $^{ab}$ | 28.9 $^{a}$ | 1.80 $^{a}$ | 16.6 $^{b}$ | 267 $^{a}$ | 16.2 $^{a}$ |
| | McMurray | 480 $^{a}$ | 27.9 $^{a}$ | 1.75 $^{a}$ | 17.2 $^{ab}$ | 275 $^{a}$ | 16.0 $^{a}$ |
| | Anzac | 477 $^{ab}$ | 25.2 $^{a}$ | 1.68 $^{a}$ | 19.3 $^{a}$ | 292 $^{a}$ | 15.9 $^{a}$ |
| *Rubus chamaemorus* | Mildred | 471 $^{c}$ | 27.6 $^{b}$ | 2.52 $^{a}$ | 17.0 $^{bc}$ | 189 $^{c}$ | 10.4 $^{b}$ |
| | JPH4 | 491 $^{b}$ | 29.5 $^{a}$ | 1.85 $^{b}$ | 16.8 $^{c}$ | 263 $^{b}$ | 15.4 $^{a}$ |
| | McKay | 486 $^{b}$ | 26.6 $^{b}$ | 1.67 $^{cd}$ | 18.1 $^{b}$ | 296 $^{ab}$ | 16.0 $^{a}$ |
| | McMurray | 492 $^{b}$ | 23.0 $^{c}$ | 1.58 $^{d}$ | 21.4 $^{a}$ | 313 $^{a}$ | 14.8 $^{a}$ |
| | Anzac | 495 $^{a}$ | 27.1 $^{ab}$ | 1.80 $^{bc}$ | 18.0 $^{bc}$ | 272 $^{b}$ | 15.0 $^{a}$ |

Table S6. Median tissue concentrations of C, N, and S, and C:N, C:S, and N:S ratios for the August sampling dates for 2010, 2013, and 2015 only. For each parameter and each species, medians with the same letter superscript do not differ significantly ($p < 0.05$) per Friedman's test (sampling date as the blocked factor); *a posteriori* comparisons made using Tukey's Honestly Significant Difference Test (α = 0.05) (Pereira et al. 2015).

| Species | Site | C (mg/g) | N (mg/g) | S (mg/g) | C:N | C:S | N:S |
|---|---|---|---|---|---|---|---|
| *Evernia mesomorpha* | Mildred | $411^{c}$ | $10.8^{a}$ | $1.72^{a}$ | $37.7^{d}$ | $247^{c}$ | $6.5^{a\text{-}c}$ |
| | JPH4 | $422^{b}$ | $9.1^{b}$ | $1.42^{ab}$ | $45.9^{c}$ | $316^{bc}$ | $6.4^{c}$ |
| | McKay | $424^{bc}$ | $8.8^{bc}$ | $1.26^{bc}$ | $48.1^{bc}$ | $331^{b}$ | $7.2^{bc}$ |
| | McMurray | $444^{a}$ | $7.4^{c}$ | $1.05^{d}$ | $59.3^{a}$ | $420^{a}$ | $7.4^{ab}$ |
| | Anzac | $452^{a}$ | $8.5^{bc}$ | $1.15^{cd}$ | $53.2^{ab}$ | $391^{a}$ | $7.4^{a}$ |
| *Cladonia mitis* | Mildred | $414^{abc}$ | $5.5^{a}$ | $0.69^{a}$ | $73.7^{a}$ | $582^{b}$ | $7.6^{b}$ |
| | JPH4 | $407^{c}$ | $5.2^{a}$ | $0.59^{ab}$ | $80.0^{a}$ | $690^{b}$ | $8.6^{b}$ |
| | McKay | $403^{bc}$ | $4.7^{a}$ | $0.56^{bc}$ | $86.5^{a}$ | $757^{b}$ | $8.6^{b}$ |
| | McMurray | $440^{a}$ | $4.6^{a}$ | $0.47^{d}$ | $94.3^{a}$ | $945^{a}$ | $10.3^{a}$ |
| | Anzac | $425^{ab}$ | $4.8^{a}$ | $0.48^{cd}$ | $91.0^{a}$ | $881^{a}$ | $9.2^{ab}$ |
| *Sphagnum fuscum* | Mildred | $441^{c}$ | $13.6^{a}$ | $1.78^{a}$ | $33.0^{a}$ | $245^{c}$ | $7.1^{c}$ |
| | JPH4 | $446^{b}$ | $14.6^{a}$ | $1.50^{b}$ | $31.0^{a}$ | $298^{bc}$ | $8.7^{bc}$ |
| | McKay | $466^{a}$ | $14.3^{a}$ | $1.55^{b}$ | $32.6^{a}$ | $307^{b}$ | $9.7^{ab}$ |
| | McMurray | $458^{a}$ | $12.5^{a}$ | $1.01^{c}$ | $36.1^{a}$ | $455^{a}$ | $11.9^{a}$ |
| | Anzac | $461^{a}$ | $12.4^{a}$ | $1.18^{c}$ | $37.8^{a}$ | $389^{a}$ | $10.3^{ab}$ |
| *Sphagnum capillifolum* | Mildred | $436^{c}$ | $12.0^{ab}$ | $1.23^{a}$ | $36.2^{a}$ | $357^{c}$ | $9.8^{b}$ |
| | JPH4 | $445^{d}$ | $13.0^{b}$ | $1.40^{a}$ | $34.2^{ab}$ | $318^{c}$ | $8.4^{c}$ |
| | McKay | $466^{bc}$ | $14.7^{a}$ | $1.40^{ab}$ | $30.4^{b}$ | $328^{bc}$ | $11.9^{a}$ |
| | McMurray | $454^{b}$ | $12.1^{b}$ | $1.10^{bc}$ | $36.6^{a}$ | $436^{ab}$ | $11.1^{a}$ |
| | Anzac | $459^{bc}$ | $13.3^{ab}$ | $1.10^{a}$ | $34.4^{ab}$ | $427^{a}$ | $11.7^{a}$ |
| *Picea mariana* | Mildred | $514^{a}$ | $8.5^{a}$ | $1.02^{a}$ | $61.6^{b}$ | $508^{c}$ | $7.9^{b}$ |
| | JPH4 | $506^{c}$ | $7.8^{ab}$ | $0.93^{a}$ | $64.8^{a}$ | $541^{c}$ | $8.4^{b}$ |
| | McKay | $507^{bc}$ | $6.9^{b}$ | $0.80^{b}$ | $73.3^{ab}$ | $625^{b}$ | $8.6^{b}$ |
| | McMurray | $511^{b}$ | $7.4^{ab}$ | $0.67^{c}$ | $69.4^{ab}$ | $768^{a}$ | $11.1^{a}$ |
| | Anzac | $506^{bc}$ | $6.8^{b}$ | $0.78^{bc}$ | $74.6^{a}$ | $652^{ab}$ | $9.0^{ab}$ |
| *Rhododendron groenlandicum* | Mildred | $525^{c}$ | $15.7^{a}$ | $1.25^{a}$ | $33.0^{a}$ | $427^{b}$ | $12.5^{b}$ |
| | JPH4 | $546^{a}$ | $15.6^{a}$ | $1.26^{a}$ | $34.6^{a}$ | $437^{b}$ | $12.2^{b}$ |
| | McKay | $523^{ab}$ | $16.2^{a}$ | $1.21^{ab}$ | $32.6^{a}$ | $449^{ab}$ | $14.0^{ab}$ |
| | McMurray | $525^{b}$ | $15.6^{a}$ | $1.02^{b}$ | $33.5^{a}$ | $514^{a}$ | $15.3^{a}$ |

| | | | | | | | |
|---|---|---|---|---|---|---|---|
| | Anzac | $533^{ab}$ | $16.5^{a}$ | $1.26^{a}$ | $32.8^{a}$ | $432^{ab}$ | $13.6^{ab}$ |
| *Vaccinium oxycoccos* | Mildred | $490^{a}$ | $13.2^{a}$ | $1.24^{a}$ | $37.0^{c}$ | $391^{c}$ | $10.7^{a}$ |
| | JPH4 | $503^{a}$ | $14.7^{a}$ | $1.24^{a}$ | $33.5^{c}$ | $401^{c}$ | $11.2^{a}$ |
| | McKay | $501^{a}$ | $13.4^{ab}$ | $1.18^{ab}$ | $37.1^{bc}$ | $424^{bc}$ | $12.2^{a}$ |
| | McMurray | $501^{a}$ | $12.8^{bc}$ | $1.09^{ab}$ | $39.5^{ab}$ | $458^{ab}$ | $11.7^{a}$ |
| | Anzac | $504^{a}$ | $12.3^{c}$ | $1.05^{b}$ | $41.0^{a}$ | $474^{a}$ | $11.4^{a}$ |
| *Vaccinium vitis-idaea* | Mildred | $512^{b}$ | $9.4^{ab}$ | $1.46^{b}$ | $54.3^{a}$ | $349^{a}$ | $6.3^{a}$ |
| | JPH4 | $520^{ab}$ | $10.9^{a}$ | $1.44^{ab}$ | $47.9^{a}$ | $365^{ab}$ | $7.2^{a}$ |
| | McKay | $512^{a}$ | $10.6^{ab}$ | $1.52^{a}$ | $49.0^{a}$ | $354^{b}$ | $6.7^{a}$ |
| | McMurray | $508^{ab}$ | $9.8^{b}$ | $1.43^{ab}$ | $52.7^{a}$ | $371^{ab}$ | $6.6^{a}$ |
| | Anzac | $523^{ab}$ | $9.9^{ab}$ | $1.58^{ab}$ | $51.4^{a}$ | $334^{ab}$ | $7.1^{a}$ |
| *Maianthemum trifolia* | Mildred | $448^{c}$ | $28.8^{a}$ | $1.99^{a}$ | $15.5^{a}$ | $222^{b}$ | $13.7^{a}$ |
| | JPH4 | $473^{bc}$ | $27.2^{a}$ | $1.78^{a}$ | $17.4^{a}$ | $265^{ab}$ | $16.7^{a}$ |
| | McKay | $475^{abc}$ | $29.4^{a}$ | $1.80^{a}$ | $16.3^{a}$ | $265^{ab}$ | $15.4^{a}$ |
| | McMurray | $480^{ab}$ | $27.1^{a}$ | $1.65^{a}$ | $17.8^{a}$ | $289^{ab}$ | $17.4^{a}$ |
| | Anzac | $484^{a}$ | $25.2^{a}$ | $1.63^{a}$ | $19.3^{a}$ | $299^{a}$ | $15.8^{a}$ |
| *Rubus chamaemorus* | Mildred | $468^{c}$ | $25.4^{b}$ | $3.38^{a}$ | $18.1^{b}$ | $137^{b}$ | $8.0^{b}$ |
| | JPH4 | $492^{a}$ | $29.7^{a}$ | $1.86^{b}$ | $16.6^{c}$ | $263^{a}$ | $14.9^{a}$ |
| | McKay | $486^{bc}$ | $27.3^{ab}$ | $1.60^{b}$ | $17.6^{bc}$ | $298^{a}$ | $17.1^{a}$ |
| | McMurray | $490^{ab}$ | $22.7^{c}$ | $1.63^{b}$ | $21.4^{a}$ | $299^{a}$ | $14.2^{a}$ |
| | Anzac | $492^{a}$ | $27.0^{ab}$ | $1.73^{b}$ | $17.8^{bc}$ | $282^{a}$ | $15.2^{a}$ |

Table S7. Regression parameters describing exponential decrease in tissue N and S concentrations and exponential increase in C:N or C:S ratios with distance from the Syncrude Mildred Lake and Suncor Base Plant upgrader stacks for the August sampling dates only for each of the eight years. Blank cells indicate that either the regression did not converge or was nonsignificant. N or S concentration = $A + Be^{-(C \times distance)}$; C:N or C:S ratios = $A + B(1-e^{-(C \times distance)})$. Regression fits performed using PROC NLIN, SAS v. 9.4. Strikethrough values are for regressions that were significant using the full data set, but are not using the August only data set. Values in bold blue font are for regressions that were nit significant using the full data set, but are using the August only data set.

| | | Tissue N regressions | | Tissue S regressions | | C:N regressions | | C:S regressions | |
|---|---|---|---|---|---|---|---|---|---|
| Species | Parameter | Syncrude; Mildred Lake | Suncor; Base Plant | Syncrude; Mildred Lake | Suncor; Base Plant | Syncrude; Mildred Lake | Suncor; Base Plant | Syncrude; Mildred Lake | Suncor; Base Plant |
| *Evernia mesomorpha* | A | 8.37 | 8.56 | 1.07 | 1.04 | 15.94 | 30.1 | 130.4 | 219.3 |
| | B | 9.77 | 14.10 | 1.49 | 0.88 | 39.17 | 25.2 | 318.5 | 294.1 |
| | C | 0.12 | 0.21 | 0.08 | 0.05 | 0.08 | 0.06 | 0.05 | 0.02 |
| | $R^2$ | 0.31 | 0.43 | 0.39 | 0.44 | 0.42 | 0.44 | 0.49 | 0.50 |
| | *p* | <0.0001 | <0.0001 | <0.0001 | <0.0001 | <0.0001 | <0.0001 | <0.0001 | <0.0001 |
| *Cladonia mitis* | A | 4.80 | 4.85 | 0.49 | 0.48 | 40.47 | 67.1 | 374.9 | 529.3 |
| | B | 2.33 | 3.48 | 0.37 | 0.31 | 53.48 | 26.4 | 547.1 | 447.8 |
| | C | 0.11 | 0.21 | 0.05 | 0.04 | 0.11 | 0.09 | 0.05 | 0.03 |
| | $R^2$ | 0.05 | 0.07 | 0.29 | 0.29 | 0.09 | 0.11 | 0.37 | 0.34 |
| | *p* | 0.0103 | 0.0020 | <0.0001 | <0.0001 | <0.0001 | <0.0001 | <0.0001 | <0.0001 |
| *Sphagnum fuscum* | A | 12.63 | 12.34 | 1.07 | 1.06 | 26.29 | 31.0 | 46.7 | 193.9 |
| | B | 3.46 | 1.79 | 1.54 | 1.08 | 12.48 | 7.4 | 396.3 | 281.2 |
| | C | 0.08 | 0.03 | 0.07 | 0.05 | 0.08 | 0.03 | 0.06 | 0.03 |
| | $R^2$ | 0.05 | 0.04 | 0.38 | 0.41 | 0.09 | 0.10 | 0.52 | 0.51 |
| | *p* | 0.0141 | 0.0293 | <0.0001 | <0.0001 | <0.0001 | 0.0002 | <0.0001 | <0.0001 |
| *Sphagnum capillifolium* | A | | | 1.05 | 1.00 | ***29.54*** | ~~34.6~~ | 96.8 | 269.0 |
| | B | | | 1.15 | 0.69 | ***9.16*** | ~~3.0~~ | 355.7 | 213.7 |
| | C | | | 0.08 | 0.04 | ***0.05*** | ~~0.04~~ | 0.08 | 0.03 |
| | $R^2$ | | | 0.20 | 0.21 | ***0.05*** | ~~0.01~~ | 0.26 | 0.26 |
| | *p* | | | <0.0001 | <0.0001 | ***0.0175*** | ~~0.0141~~ | <0.0001 | <0.0001 |

| | | | | | | | | | |
|---|---|---|---|---|---|---|---|---|---|
| *Picea mariana* | A | ***7.09*** | ***7.08*** | ***0.73*** | 0.72 | | 39.4 | | 411.7 |
| | B | ***72.51*** | ***4.04*** | ***1.79*** | 0.45 | | 34.5 | | 327.5 |
| | C | ***0.32*** | ***0.16*** | ***0.15*** | 0.07 | | 0.15 | | 0.06 |
| | $R^2$ | ***0.09*** | ***0.12*** | ***0.30*** | 0.32 | | 0.12 | | 0.32 |
| | *p* | ***0.0002*** | ***<0.0001*** | ***<0.0001*** | <0.0001 | | <0.0001 | | <0.0001 |
| *Vaccinium oxycoccos* | A | 11.80 | 8.43 | 1.00 | 0.70 | 33.24 | ~~37.7~~ | 367.6 | ~~384.7~~ |
| | B | 3.36 | 4.86 | 0.41 | 0.66 | 12.19 | ~~15.2~~ | 164.1 | ~~136.7~~ |
| | C | 0.04 | 0.01 | 0.02 | 0.01 | 0.03 | ~~0.01~~ | 0.02 | ~~0.04~~ |
| | $R^2$ | 0.12 | 0.08 | 0.17 | 0.18 | 0.12 | ~~0.11~~ | 0.22 | ~~0.16~~ |
| | *p* | <0.0001 | <0.0001 | <0.0001 | <0.0001 | <0.0001 | ~~<0.0001~~ | <0.0001 | ~~<0.0001~~ |
| *Vaccinium vitis-idaea* | A | 10.07 | ~~9.10~~ | | | 44.97 | ~~48.5~~ | | |
| | B | 1.54 | ~~1.99~~ | | | 7.61 | ~~7.3~~ | | |
| | C | 0.06 | ~~0.01~~ | | | 0.06 | ~~0.01~~ | | |
| | $R^2$ | 0.04 | ~~0.03~~ | | | 0.04 | ~~0.03~~ | | |
| | *p* | 0.0242 | ~~0.0002~~ | | | 0.0250 | ~~<0.0001~~ | | |
| *Rubus chamaemorus* | A | 25.18 | 25.37 | 1.68 | 1.70 | 8.04 | 14.4 | | |
| | B | 22.54 | 6.24 | 5.04 | 22.62 | 11.74 | 5.2 | | |
| | C | 0.14 | 0.08 | 0.17 | 0.35 | 0.24 | 0.08 | | |
| | $R^2$ | 0.20 | 0.15 | 0.10 | 0.41 | 0.05 | 0.19 | | |
| | *p* | <0.0001 | <0.0001 | <0.0001 | <0.0001 | <0.0001 | <0.0001 | | |

Table S8. Regression parameters describing exponential decrease in tissue N and S concentrations and exponential increase in C:N or C:S ratios with distance from the Syncrude Mildred Lake and Suncor Base Plant upgrader stacks for the August sampling dates for 2010, 2013, and 2015 only. Blank cells indicate that either the regression did not converge or was nonsignificant. N or S concentration = $A + Be^{-(C \text{ x } distance)}$; C:N or C:S ratios = $A + B(1-e^{-(C \text{ x } distance)})$. Regression fits performed using PROC NLIN, SAS v. 9.4. Data from the spring collections for which N concentrations peaked (Fig. 1) were excluded from the analysis. Strikethrough values are for regressions that were significant using the full data set, but are not using the three year August only data set. Values in bold blue font are for regressions that were nit significant using the full data set, but are using the three year August only data set.

| | | Tissue N regressions | | Tissue S regressions | | C:N regressions | | C:S regressions | |
|---|---|---|---|---|---|---|---|---|---|
| Species | Parameter | Syncrude; Mildred Lake | Suncor; Base Plant | Syncrude; Mildred Lake | Suncor; Base Plant | Syncrude; Mildred Lake | Suncor; Base Plant | Syncrude; Mildred Lake | Suncor; Base Plant |
| *Evernia mesomorpha* | A | 8.0 | 8.3 | 1.1 | 1.1 | 16.4 | 25.1 | 63.7 | 195.9 |
| | B | 8.7 | 19.5 | 1.8 | 1.0 | 440.4 | 30.7 | 366.4 | 254.6 |
| | C | 0.11 | 0.24 | 0.10 | 0.08 | 0.08 | 0.08 | 0.06 | 0.04 |
| | $R^2$ | 0.32 | 0.47 | 0.44 | 0.51 | 0.41 | 0.44 | 0.53 | 0.52 |
| | *p* | <0.0001 | <0.0001 | <0.0001 | <0.0001 | <0.0001 | <0.0001 | <0.0001 | <0.0001 |
| *Cladonia mitis* | A | 2.6 | ~~5.1~~ | 0.5 | 0.5 | 69.5 | 73.7 | 310.6 | 496.9 |
| | B | 3.0 | ~~3.3~~ | 0.3 | 0.3 | 29.4 | 40.8 | 613.2 | 448.4 |
| | C | 0.01 | ~~0.16~~ | 0.04 | 0.04 | 0.03 | 0.01 | 0.06 | 0.04 |
| | $R^2$ | 0.12 | ~~0.12~~ | 0.32 | 0.31 | 0.19 | 0.20 | 0.38 | 0.37 |
| | *p* | <0.0001 | ~~<0.0001~~ | <0.0001 | <0.0001 | <0.0001 | 0.0007 | <0.0001 | <0.0001 |
| *Sphagnum fuscum* | A | 10.7 | ~~11.68~~ | 1.1 | 1.0 | 29.7 | ~~32.9~~ | 37.7 | 183.2 |
| | B | 4.0 | ~~1.44~~ | 1.3 | 0.9 | 10.9 | ~~7.4~~ | 395.3 | 265.0 |
| | C | 0.01 | ~~0.02~~ | 0.06 | 0.04 | 0.02 | ~~0.03~~ | 0.06 | 0.04 |
| | $R^2$ | 0.11 | ~~0.02~~ | 0.57 | 0.57 | 0.18 | ~~0.09~~ | 0.59 | 0.56 |
| | *p* | 0.0182 | ~~0.0004~~ | <0.0001 | <0.0001 | 0.0013 | ~~<0.0001~~ | <0.0001 | <0.0001 |
| *Sphagnum capillifolium* | A | | | 1.1 | 0.9 | ***31.5*** | ~~34.6~~ | 55.9 | 283.8 |
| | B | | | 1.1 | 0.7 | ***7.3*** | ~~3.0~~ | 384.2 | 198.3 |
| | C | | | 0.07 | 0.02 | ***0.02*** | ~~0.04~~ | 0.09 | 0.03 |
| | $R^2$ | | | 0.14 | 0.14 | ***0.09*** | ~~0.01~~ | 0.19 | 0.17 |

| | | | | | | | | | |
|---|---|---|---|---|---|---|---|---|---|
| | *p* | | | 0.0066 | 0.0088 | ***0.0458*** | ~~0.0141~~ | 0.0013 | <0.0001 |
| *Picea mariana* | A | ***7.0*** | ***7.0*** | | 0.7 | | 14.2 | | 360.5 |
| | B | ***14.6*** | ***14.5*** | | 0.7 | | 59.0 | | 375.1 |
| | C | ***0.21*** | ***0.29*** | | 0.09 | | 0.22 | | 0.06 |
| | $R^2$ | ***0.11*** | ***0.22*** | | 0.05 | | 0.15 | | 0.46 |
| | *p* | ***0.0207*** | ***0.0002*** | | <0.0001 | | 0.0046 | | <0.0001 |
| *Vaccinium oxycoccos* | A | ~~11.6~~ | ~~8.4~~ | ~~1.0~~ | 1.1 | ~~32.8~~ | ~~37.7~~ | ~~159.9~~ | 371.2 |
| | B | ~~2.9~~ | ~~4.9~~ | ~~0.6~~ | 0.3 | ~~11.8~~ | ~~15.2~~ | ~~288.7~~ | 95.0 |
| | C | ~~0.05~~ | ~~0.01~~ | ~~0.08~~ | 0.03 | ~~0.05~~ | ~~0.01~~ | ~~0.12~~ | 0.03 |
| | $R^2$ | ~~0.09~~ | ~~0.08~~ | ~~0.15~~ | 0.08 | ~~0.12~~ | ~~0.11~~ | ~~0.09~~ | 0.11 |
| | *p* | ~~<0.0001~~ | ~~<0.0001~~ | ~~<0.0001~~ | <0.0001 | ~~<0.0001~~ | ~~<0.0001~~ | ~~0.0376~~ | 0.0193 |
| *Vaccinium vitis-idaea* | A | ~~10.0~~ | ~~9.1~~ | | | ~~44.3~~ | *48.5* | | |
| | B | ~~2.0~~ | ~~2.0~~ | | | ~~8.5~~ | *7.3* | | |
| | C | ~~0.05~~ | ~~0.01~~ | | | ~~0.05~~ | *0.01* | | |
| | $R^2$ | ~~0.05~~ | ~~0.03~~ | | | ~~0.05~~ | *0.03* | | |
| | *p* | ~~<0.0001~~ | ~~0.0002~~ | | | ~~<0.0001~~ | *<0.0001* | | |
| *Rubus chamaemorus* | A | 25.1 | 25.2 | 1.7 | 1.7 | 12.1 | 15.9 | | |
| | B | 13.5 | 4.6 | 5.4 | 101.7 | 17.6 | 3.6 | | |
| | C | 0.10 | 0.05 | 0.15 | 0.51 | 0.07 | 0.05 | | |
| | $R^2$ | 0.22 | 0.12 | 0.19 | 0.70 | 0.26 | 0.15 | | |
| | *p* | 0.0002 | 0.0154 | 0.0015 | <0.0001 | <0.0001 | <0.0001 | | |

Table S9. Correlations (Kendall's Tau) between tissue N or S concentrations, C:N ratios, and C:S ratios and time (years) for the August sampling dates only for each of the eight years. Correlations that were significant (one-sided test for positive correlation) using the full data set and remain so using the August only data set are in bold, black, italicized font. Red font indicates a correlation was significant using the full data set, but not significant using the August only data. Blue bold italicized font indicates a correlation was not significant using the full data set, but is significant using the August only data.

| | | | Site | | | | |
|---|---|---|---|---|---|---|---|
| Species | Element or Ratio | | Mildred | JPH4 | McKay | McMurray | Anzac |
| *Cladonia mitis* | N | *τ* | -0.0079 | -0.1397 | -0.1426 | -0.2848 | 0.0286 |
| | | *p* | 0.9595 | 0.2623 | 0.2221 | 0.0135 | 0.8120 |
| | C:N | *τ* | -0.0794 | 0.1306 | 0.1097 | 0.2355 | -0.0874 |
| | | *p* | 0.6115 | 0.2941 | 0.3472 | 0.0408 | 0.4674 |
| | S | *τ* | ***-0.4541*** | 0.2082 | ***-0.4520*** | ***-0.4873*** | ***-0.1751*** |
| | | *p* | 0038 | 0.0654 | ***<0.0001*** | ***<0.0001*** | ***0.0160*** |
| | C:S | *τ* | ***0.3572*** | -0.1843 | ***0.3604*** | ***0.4658*** | 0.1827 |
| | | *p* | ***0.0223*** | 0.1388 | ***0.0020*** | ***<0.0001*** | 0.1287 |
| *Evernia mesomorpha* | N | *τ* | -0.2556 | 0.0376 | -0.0420 | 0.0983 | -0.0812 |
| | | *p* | 0.0943 | 0.7628 | 0.7154 | 0.3995 | 0.4865 |
| | C:N | *τ* | -0.1314 | -0.1592 | 0.0054 | -0.1610 | 0.0499 |
| | | *p* | 0.3895 | 0.2009 | 0.9625 | 0.1677 | 0.6691 |
| | S | *τ* | ***-0.6218*** | -0.1113 | ***-0.5265*** | ***-0.2812*** | ***-0.3043*** |
| | | *p* | ***<0.0001*** | 0.3727 | ***<0.0001*** | ***0.0176*** | ***0.0093*** |
| | C:S | *τ* | ***0.5039*** | 0.0018 | ***0.4890*** | ***0.2327*** | ***0.2949*** |
| | | *p* | ***0.0010*** | 0.9885 | ***<0.0001*** | ***<.0492*** | ***0.0115*** |
| *Sphagnum capillifolium* | N | *τ* | -0.0714 | -0.3457 | -0.5027 | -0.3611 | -0.2437 |
| | | *p* | 0.6475 | 0.0063 | <0.0001 | 0.0036 | 0.0492 |
| | C:N | *τ* | 0.0317 | 0.2714 | 0.4276 | 0.2275 | 0.0658 |
| | | *p* | 0.8390 | 0.0319 | 0.0006 | 0.0663 | 0.5954 |
| | S | *τ* | ***-0.4250*** | ***-0.5375*** | ***-0.5123*** | ***-0.4661*** | ***-0.4323*** |
| | | *p* | ***0.0055*** | ***<0.0001*** | ***<0.0001*** | ***0.0001*** | ***<0.0001*** |
| | C:S | *τ* | ***0.4128*** | ***0.4915*** | ***0.4240*** | ***0.3768*** | ***0.3338*** |
| | | *p* | ***0.0083*** | ***0.0001*** | ***0.0007*** | ***0.0023*** | ***0.0080*** |
| *Sphagnum fuscum* | N | *τ* | ***0.6145*** | 0.1056 | 0.0343 | 0.0214 | ***0.2832*** |
| | | *p* | ***<0.0001*** | 0.3965 | 0.7694 | 0.8547 | ***0.0140*** |
| | C:N | *τ* | ***-0.6135*** | -0.1414 | 0.0128 | -0.0014 | ***-0.2842*** |
| | | *p* | ***<0.0001*** | 0.2562 | 0.9125 | 0.9903 | ***0.0135*** |

| | | | | | | | |
|---|---|---|---|---|---|---|---|
| | S | *τ* | -0.2346 | ***-0.2539*** | ***-0.4513*** | ***-0.4804*** | -0.1783 |
| | | *p* | 0.1339 | ***0.0579*** | ***0.0003*** | ***<0.0001*** | 0.1269 |
| | C:S | *τ* | ***0.2858*** | 0.1883 | ***0.4652*** | ***0.4886*** | 0.1781 |
| | | *p* | ***0.0674*** | 0.1577 | ***0.0002*** | ***<0.0001*** | 0.1270 |
| *Picea mariana* | N | *τ* | -0.1314 | -0.0241 | -0.2626 | -0.2923 | -0.2087 |
| | | *p* | 0.3895 | 0.0567 | 0.0246 | 0.0111 | 0.0701 |
| | C:N | *τ* | 0.1314 | 0.2866 | 0.2920 | 0.3248 | 0.2788 |
| | | *p* | 0.3895 | 0.0235 | 0.0123 | 0.0048 | 0.0154 |
| | S | *τ* | 0.0514 | ***-0.2871*** | ***-0.2261*** | ***-0.4022*** | ***-0.3666*** |
| | | *p* | 0.7377 | ***0.0234*** | ***0.0536*** | ***0.0005*** | ***0.0015*** |
| | C:S | *τ* | -0.0366 | ***0.2638*** | ***0.2250*** | ***0.4195*** | ***0.3681*** |
| | | *p* | 0.8111 | ***0.0370*** | ***0.0440*** | ***<.0003*** | ***0.0014*** |
| *Rhododendron groenlandicum* | N | *τ* | 0.0476 | -0.4849 | -0.5407 | -0.2304 | ***0.1967*** |
| | | *p* | 0.7605 | <0.0001 | <0.0001 | 0.0456 | ***0.0071*** |
| | C:N | *τ* | -0.2540 | -0.4240 | 0.3681 | 0.1029 | ***-0.3627*** |
| | | *p* | 0.1040 | 0.0007 | 0.0014 | 0.3715 | ***0.0016*** |
| | S | *τ* | ***-0.3261*** | 0.2316 | -0.1874 | -0.0691 | 0.0285 |
| | | *p* | ***0.0372*** | 0.0637 | 0.1046 | 0.5487 | 0.8049 |
| | C:S | *τ* | 0.2381 | -0.2988 | 0.1028 | -0.0027 | -0.0514 |
| | | *p* | 0.1175 | 0.0164 | 0.3715 | 0.9812 | 0.6550 |
| *Vaccinium oxycoccos* | N | *τ* | -0.0079 | 0.1270 | 0.1694 | 0.1483 | ***0.4667*** |
| | | *p* | 0.9595 | 0.3075 | 0.1416 | 0.2041 | ***<0.0001*** |
| | C:N | *τ* | 0.0159 | -0.1736 | ***-0.1922*** | -0.1068 | ***-0.5413*** |
| | | *p* | ***0.9191*** | 0.1633 | ***0.0950*** | 0.3598 | ***<0.0001*** |
| | S | *τ* | -0.2346 | ***-0.2222*** | -0.0488 | 0.0943 | 0.1143 |
| | | *p* | 0.1339 | ***0.0844*** | 0.6720 | 0.4202 | 0.3230 |
| | C:S | *τ* | ***0.2699*** | 0.1855 | 0.1055 | -0.0584 | 0.0650 |
| | | *p* | ***0.0841*** | 0.1490 | 0.3591 | 0.6167 | 0.5725 |
| *Vaccinium vitis-idaea* | N | *τ* | -0.2748 | -0.4391 | -0.1314 | -0.2778 | 0.1786 |
| | | *p* | 0.0729 | 0.0004 | 0.2540 | 0.0189 | 0.1207 |
| | C:N | *τ* | 0.2483 | 0.3918 | 0.0920 | 0.2147 | ***-0.2409*** |
| | | *p* | 0.1041 | 0.0016 | 0.4240 | 0.0696 | ***0.0364*** |
| | S | *τ* | ***-0.2597*** | ***-0.2165*** | -0.0500 | -0.1029 | 0.0869 |
| | | *p* | ***0.0896*** | ***0.0820*** | 0.6690 | 0.3792 | 0.4516 |
| | C:S | *τ* | 0.1241 | 0.2022 | 0.0014 | 0.0345 | -0.1110 |
| | | *p* | 0.4164 | 0.1044 | 0.9903 | 0.7704 | 0.3350 |
| *Maianthemum trifolium* | N | *τ* | 0.0721 | 0.3535 | -0.3651 | -0.1659 | 0.0160 |
| | | *p* | 0.7349 | 0.2008 | 0.0020 | 0.1733 | 0.9440 |

| | | | | | | | |
|---|---|---|---|---|---|---|---|
| | C:N | *τ* | -0.1202 | -0.2357 | 0.3618 | 0.1659 | -0.0160 |
| | | *p* | 0.5725 | 0.3938 | 0.0022 | 0.1733 | 0.9440 |
| | S | *τ* | -0.1405 | -0.0589 | ***-0.3714*** | ***-0.2166*** | ***-0.4789*** |
| | | *p* | 0.2590 | 0.8312 | ***0.0017*** | ***0.0760*** | ***0.0747*** |
| | C:S | *τ* | 0.2164 | 0.1178 | ***0.3613*** | ***0.2296*** | ***0.4789*** |
| | | *p* | 0.3097 | 0.6698 | ***0.0026*** | ***0.0595*** | ***0.0747*** |
| *Rubus chamaemorus* | N | *τ* | 0.0416 | -0.4900 | -0.2030 | -0.4764 | -0.3279 |
| | | *p* | 0.8385 | <0.0001 | 0.0778 | 0.0001 | 0.0050 |
| | C:N | *τ* | -0.0416 | 0.4915 | 0.0514 | 0.4764 | 0.2949 |
| | | *p* | 0.8385 | 0.0001 | 0.6550 | <0.0001 | 0.0115 |
| | S | *τ* | 0.2081 | ***-0.5413*** | ***-0.4746*** | -0.1585 | -0.1202 |
| | | *p* | 0.3083 | ***<0.0001*** | ***<0.0001*** | 0.1752 | 0.3100 |
| | C:S | *τ* | -0.1456 | ***0.5598*** | ***0.4358*** | 0.1553 | 0.0886 |
| | | *p* | 0.4757 | ***<0.0001*** | ***0.0002*** | 0.1832 | 0.4540 |

Table S10. Correlations (Kendall's Tau) between tissue N or S concentrations, C:N ratios, and C:S ratios and time (years) for the August sampling dates for 2010, 2013, and 2015 only. Correlations that were significant (one-sided test for positive correlation) using the full data set and remain so using the three-year August only data set are in bold, black, italicized font. Red font indicates a correlation was significant using the full data set, but not significant using the three year August only data. Blue bold italicized font indicates a correlation was not significant using the full data set, but is significant using the three year August only data.

| | | | Site | | | | |
|---|---|---|---|---|---|---|---|
| Species | Element or Ratio | | Mildred | JPH4 | McKay | McMurray | Anzac |
| *Cladonia mitis* | N | $\tau$ | -0.5665 | -0.2817 | -0.4169 | -0.5209 | ***0.4868*** |
| | | *p* | 0.0472 | 0.1866 | 0.0506 | 0.0150 | ***0.0230*** |
| | C:N | $\tau$ | 0.5068 | 0.3718 | 0.3268 | 0.4395 | ***-0.6198*** |
| | | *p* | 0.0758 | 0.0813 | 0.1255 | 0.0394 | ***0.0037*** |
| | S | $\tau$ | -0.2111 | -0.0910 | ***-0.3887*** | ***-0.4785*** | 0.2366 |
| | | *p* | 0.4633 | 0.6720 | ***0.0716*** | ***0.0098*** | 0.2673 |
| | C:S | $\tau$ | 0.0894 | 0.1014 | 0.1465 | ***0.5071*** | -0.1690 |
| | | *p* | 0.7510 | 0.6345 | 0.4922 | ***0.0224*** | 0.4281 |
| *Evernia mesomorpha* | N | $\tau$ | -0.5068 | ***0.5296*** | 0.3493 | 0.1690 | ***0.4291*** |
| | | *p* | 0.0758 | ***0.0130*** | 0.1015 | 0.4281 | ***0.0533*** |
| | C:N | $\tau$ | 0.3280 | ***-0.6198*** | -0.3493 | -0.2141 | ***-0.4031*** |
| | | *p* | 0.2506 | ***0.0037*** | 0.1015 | 0.3155 | ***0.0695*** |
| | S | $\tau$ | ***-0.5068*** | 0.3170 | ***-0.4416*** | -0.2471 | 0.2498 |
| | | *p* | ***0.0758*** | 0.1387 | ***<0.0001*** | 0.2659 | 0.2647 |
| | C:S | $\tau$ | ***0.5068*** | -0.5296 | ***0.3944*** | 0.1690 | -0.2471 |
| | | *p* | 0.0758 | 0.0130 | ***0.0645*** | 0.4465 | 0.0629 |
| *Sphagnum capillifolium* | N | $\tau$ | -0.7454 | -0.2366 | -0.5747 | -0.3661 | -0.1690 |
| | | *p* | 0.0090 | 0.2673 | 0.0071 | 0.1007 | 0.4281 |
| | C:N | $\tau$ | 0.7454 | 0.2366 | 0.3268 | 0.1950 | 0.0113 |
| | | *p* | 0.0009 | 0.2673 | 0.1255 | 0.3798 | 0.9579 |
| | S | $\tau$ | ***-0.7538*** | ***-0.7586*** | ***-0.7099*** | ***-0.4576*** | ***-0.5622*** |
| | | *p* | ***0.0088*** | ***0.0004*** | ***0.0009*** | ***0.0402*** | ***0.0117*** |
| | C:S | $\tau$ | ***0.7454*** | ***0.6874*** | ***0.5522*** | ***0.4031*** | ***0.5071*** |
| | | *p* | ***0.0009*** | ***0.0013*** | ***0.0096*** | ***0.0695*** | ***0.0224*** |
| *Sphagnum fuscum* | N | $\tau$ | ***0.6332*** | 0.2592 | -0.1812 | 0.0390 | ***0.6907*** |
| | | *p* | ***0.0278*** | 0.2244 | 0.3975 | 0.8606 | ***0.0013*** |
| | C:N | $\tau$ | ***-0.6261*** | ***-0.3718*** | 0.2592 | -0.0910 | ***-0.6874*** |
| | | *p* | ***0.0283*** | ***0.0813*** | 0.2244 | 0.6819 | ***0.0013*** |

| | | | | | | | |
|---|---|---|---|---|---|---|---|
| | S | *τ* | -0.2981 | -0.1766 | -0.2963 | ***-0.7191*** | -0.1430 |
| | | *p* | 0.3272 | 0.4958 | 0.2572 | ***0.0013*** | <0.5195 |
| | C:S | *τ* | 0.4472 | 0.0875 | 0.3875 | ***0.6891*** | 0.1950 |
| | | *p* | 0.1416 | 0.7341 | 0.1385 | ***0.0019*** | 0.3798 |
| *Picea mariana* | N | *τ* | -0.7454 | -0.1690 | -0.3397 | -0.6874 | -0.4665 |
| | | *p* | 0.0090 | 0.4465 | 0.1127 | 0.0013 | 0.0300 |
| | C:N | *τ* | 0.7454 | 0.0910 | 0.2366 | 0.6423 | 0.4846 |
| | | *p* | 0.0090 | 0.3819 | 0.2673 | 0.0026 | 0.0231 |
| | S | *τ* | ***-0.4880*** | ***-0.4076*** | -0.1944 | ***-0.5972*** | ***-0.4916*** |
| | | *p* | ***0.0927*** | ***0.0688*** | 0.3673 | ***0.0051*** | ***0.0227*** |
| | C:S | *τ* | 0.3876 | 0.3511 | 0.1240 | ***0.5747*** | ***0.5071*** |
| | | *p* | 0.1745 | 0.1139 | 0.5612 | ***0.0071*** | ***0.0174*** |
| *Rhododendron groenlandicum* | N | *τ* | 0.2382 | -0.1465 | -0.5661 | -0.3944 | 0.0113 |
| | | *p* | 0.3272 | 0.4922 | 0.0082 | 0.0645 | 0.9579 |
| | C:N | *τ* | ***-0.5217*** | 0.0113 | 0.3994 | 0.1916 | ***-0.4169*** |
| | | *p* | ***0.0864*** | 0.9579 | 0.0645 | 0.3692 | ***0.0506*** |
| | S | *τ* | 0.1512 | 0.2162 | ***-0.5831*** | -0.1359 | 0.2265 |
| | | *p* | 0.6228 | 0.3145 | ***0.0068*** | 0.5257 | 0.2902 |
| | C:S | *τ* | -0.1491 | -0.3493 | ***0.3719*** | -0.1240 | -0.3043 |
| | | *p* | 0.3242 | 0.1015 | ***0.0813*** | 0.5612 | 0.1538 |
| *Vaccinium oxycoccos* | N | *τ* | -0.6261 | 0.2141 | 0.2817 | -0.0679 | 0.2817 |
| | | *p* | 0.0283 | 0.3155 | 0.1866 | 0.7510 | 0.1866 |
| | C:N | *τ* | 0.7454 | ***-0.2817*** | -0.2817 | 0.1014 | ***-0.4260*** |
| | | *p* | 0.0090 | ***0.1866*** | 0.1866 | 0.6345 | ***0.0303*** |
| | S | *τ* | ***-0.6261*** | ***-0.4291*** | -0.2817 | 0.3493 | ***-0.4076*** |
| | | *p* | ***0.0283*** | ***0.0533*** | 0.1866 | 0.1015 | ***0.0570*** |
| | C:S | *τ* | ***0.6857*** | 0.3511 | ***0.5747*** | -0.3268 | 0.3268 |
| | | *p* | ***0.0163*** | 0.1139 | ***0.0071*** | 0.1255 | 0.1255 |
| *Vaccinium vitis-idaea* | N | *τ* | -0.3317 | -0.0563 | -0.1465 | -0.3771 | -0.0240 |
| | | *p* | 0.2492 | 0.7917 | 0.4922 | 0.0895 | 0.5612 |
| | C:N | *τ* | 0.3280 | 0.0113 | 0.0338 | 0.0910 | 0.0563 |
| | | *p* | 0.2506 | 0.9579 | 0.8741 | 0.6819 | 0.7917 |
| | S | *τ* | -0.4221 | 0.2817 | 0.2817 | 0.2604 | -0.1812 |
| | | *p* | 0.1425 | 0.1866 | 0.1866 | 0.2239 | 0.3975 |
| | C:S | *τ* | 0.1410 | -0.3493 | -0.3493 | -0.5331 | 0.1465 |
| | | *p* | 0.6015 | 0.1015 | 0.1015 | 0.0164 | 0.4922 |
| *Maianthemum trifolium* | N | *τ* | -0.2357 | 0.8165 | -0.0910 | -0.0789 | -0.5477 |
| | | *p* | 0.6547 | 0.2207 | 0.6819 | 0.7115 | 0.2786 |

| | | | | | | | |
|---|---|---|---|---|---|---|---|
| | C:N | $\tau$ | 0.2357 | 0.8165 | 0.0910 | 0.1014 | 0.5477 |
| | | $p$ | 0.6547 | 0.2207 | 0.6819 | 0.6345 | 0.2786 |
| | S | $\tau$ | -0.7071 | 0.8160 | -0.2471 | -0.1245 | -0.5477 |
| | | $p$ | 0.1797 | 0.2207 | 0.2659 | 0.5608 | 0.2786 |
| | C:S | $\tau$ | 0.7071 | -0.8160 | 0.3027 | 0.1240 | 0.5477 |
| | | $p$ | 0.1797 | 0.2207 | 0.1917 | 0.5612 | 0.2786 |
| *Rubus chamaemorus* | N | $\tau$ | -0.3651 | -0.2367 | -0.1690 | -0.5747 | -0.5071 |
| | | $p$ | 0.3545 | 0.2673 | 0.4281 | 0.0071 | 0.0174 |
| | C:N | $\tau$ | 0.1826 | 0.1916 | -0.2817 | 0.5522 | 0.2817 |
| | | $p$ | 0.6434 | 0.3692 | 0.1866 | 0.0096 | 0.1866 |
| | S | $\tau$ | 0.3651 | ***-0.5522*** | ***-0.8233*** | 0.3397 | -0.3251 |
| | | $p$ | 0.3454 | ***0.0096*** | ***0.0001*** | 0.1127 | 0.1432 |
| | C:S | $\tau$ | -0.3651 | ***0.5522*** | ***0.5296*** | -0.3268 | 0.1170 |
| | | $p$ | 0.3545 | ***0.0096*** | ***0.0130*** | 0.1255 | 0.5982 |

Table S11. Kendall's correlation coefficients (***τ***) and *p* values for tissue N or S concentrations, C:N and C:S ratios for the August sampling dates only for each of the eight years with growing season $NH_4^+$-N, $NO_3^-$-N, or $SO_4^{2-}$-S deposition (from Wieder et al. 2016b). Correlations that were significant (one-sided test for positive correlation) using the full data set and remain so using the August only data set are in bold, black, italicized font. Red font indicates a correlation was significant using the full data set, but not significant using the August only data. Blue, bold, italicized font indicates a correlation was not significant using the full data set, but is significant using the August only data.

| Species | | N concentration with growing season $NH_4^+$-N deposition | N concentration with growing season $NO_3^-$-N deposition | S concentration with growing season $SO_4^{2-}$-S deposition | C:N with growing season $NH_4^+$-N deposition | C:N with growing season $NO_3^-$-N deposition | C:S with growing season $SO_4^{2-}$-S deposition |
|---|---|---|---|---|---|---|---|
| *Cladonia mitis* | ***τ*** | -0.0141 | -0.1379 | ***0.1069*** | -0.0064 | 0.0868 | ***-0.1155*** |
| | *p* | 0.7843 | 0.075 | ***0.0388*** | 0.9013 | 0.0920 | ***0.0252*** |
| *Evernia mesomorpha* | ***τ*** | ***0.1681*** | ***0.2616*** | ***0.1084*** | ***-0.1546*** | ***-0.3092*** | ***-0.1688*** |
| | *p* | ***0.0010*** | ***<0.0001*** | ***0.0346*** | ***0.0025*** | ***<0.0001*** | ***0.0010*** |
| *Sphagnum capillifolium* | ***τ*** | -0.0832 | -0.1291 | 0.0847 | 0.0365 | 0.0607 | ***-0.1385*** |
| | *p* | 0.1199 | 0.0157 | 0.1108 | 0.4943 | 0.2560 | ***0.0097*** |
| *Sphagnum fuscum* | ***τ*** | ***0.1225*** | ***0.1496*** | ***0.1480*** | ***-0.1109*** | ***-0.1786*** | ***-0.1522*** |
| | *p* | ***0.0166*** | ***0.0034*** | ***0.0049*** | ***0.0301*** | ***0.0005*** | ***0.0038*** |
| *Picea mariana* | ***τ*** | 0.0447 | -0.0026 | ***0.1058*** | -0.0285 | 0.0037 | ***-0.0976*** |
| | *p* | 0.3823 | 0.9588 | ***0.0386*** | 0.5771 | 0.9418 | ***0.0556*** |
| *Rhododendron groenlandicum* | ***τ*** | -0.0171 | -0.0911 | -0.0347 | -0.0198 | 0.0352 | -0.0076 |
| | *p* | 0.7370 | 0.0737 | 0.4963 | 0.6969 | 0.4893 | 0.8890 |
| *Vaccinium oxycoccos* | ***τ*** | 0.0294 | ***0.2592*** | ***0.1389*** | -0.0069 | ***-0.2633*** | ***-0.1072*** |
| | *p* | 0.5651 | ***<0.0001*** | ***<0.0001*** | 0.8919 | ***<0.0001*** | ***0.0366*** |
| *Vaccinium vitis-idaea* | ***τ*** | -0.0569 | 0.0597 | -0.0950 | 0.0434 | ***-0.1113*** | 0.0537 |
| | *p* | 0.2663 | 0.2423 | 0.0630 | 0.3962 | ***0.0293*** | 0.2939 |
| *Maianthemum trifolium* | ***τ*** | -0.0294 | 0.0416 | -0.1431 | 0.0209 | -0.0608 | 0.1272 |
| | *p* | 0.6558 | 0.5275 | 0.0322 | 0.7511 | 0.3561 | 0.0576 |
| *Rubus chamaemorus* | ***τ*** | -0.1298 | 0.0220 | -0.1046 | 0.1145 | -0.0580 | 0.1019 |
| | *p* | 0.0138 | 0.6745 | 0.0480 | 0.0297 | 0.2698 | 0.0538 |

Table S12. Kendall's correlation coefficients (***τ***) and *p* values for tissue N or S concentrations, C:N and C:S ratios for the August sampling dates for 2010, 2013, and 2015 only with $NH_4^+$-N, $NO_3^-$-N, or $SO_4^{2-}$-S deposition (from Wieder et al. 2016b). Correlations that were significant (one-sided test for positive correlation) using the full data set and remain so using the three-year August only data set are in bold, black, italicized font. Red font indicates a correlation was significant using the full data set, but not significant using the three-year August only data. Blue, bold, italicized font indicates a correlation was not significant using the full data set, but is significant using the three-year August only data.

| Species | | N concentration with growing season $NH_4^+$-N deposition | N concentration with growing season $NO_3^-$-N deposition | S concentration with growing season $SO_4^{2-}$-S deposition | C:N with growing season $NH_4^+$-N deposition | C:N with growing season $NO_3^-$-N deposition | C:S with growing season $SO_4^{2-}$-S deposition |
|---|---|---|---|---|---|---|---|
| *Cladonia mitis* | ***τ*** | -0.0820 | -0.1881 | 0.0950 | 0.0414 | 0.1579 | -0.0404 |
| | *p* | 0.3289 | 0.0257 | 0.2638 | 0.6218 | 0.0610 | 0.6327 |
| *Evernia mesomorpha* | ***τ*** | ***0.2799*** | ***0.1737*** | -0.0934 | ***-0.2538*** | ***-0.2531*** | 0.0163 |
| | *p* | ***0.0010*** | ***0.0409*** | 0.2740 | ***0.0027*** | ***0.0029*** | 0.8484 |
| *Sphagnum capillifolium* | ***τ*** | -0.2659 | -0.2616 | 0.0453 | 0.1888 | 0.1427 | -0.0950 |
| | *p* | 0.0017 | 0.0021 | 0.5954 | 0.0255 | 0.0929 | 0.2648 |
| *Sphagnum fuscum* | ***τ*** | ***0.1653*** | ***0.2242*** | 0.0393 | ***-0.1660*** | ***-0.2071*** | -0.0302 |
| | *p* | ***0.0508*** | ***0.0084*** | 0.6696 | ***0.0496*** | ***0.0147*** | 0.7428 |
| *Picea mariana* | ***τ*** | -0.0246 | -0.1341 | 0.0381 | 0.0009 | 0.1338 | -0.0290 |
| | *p* | 0.7711 | 0.1149 | 0.6548 | 0.9917 | 0.1150 | 0.7317 |
| *Rhododendron groenlandicum* | ***τ*** | -0.1169 | -0.2188 | -0.0932 | -0.0316 | 0.0826 | 0.0053 |
| | *p* | 0.1670 | 0.0100 | 0.2728 | 0.7084 | 0.3307 | 0.9503 |
| *Vaccinium oxycoccos* | ***τ*** | -0.0517 | ***0.2980*** | -0.0352 | 0.0132 | ***-0.2685*** | 0.0808 |
| | *p* | 0.5384 | ***0.0004*** | 0.6776 | 0.8747 | ***0.0014*** | 0.3391 |
| *Vaccinium vitis-idaea* | ***τ*** | -0.2615 | -0.0522 | -0.2612 | 0.2099 | -0.0517 | 0.2108 |
| | *p* | 0.0020 | 0.5393 | 0.0019 | 0.0130 | 0.5428 | 0.0126 |
| *Maianthemum trifolium* | ***τ*** | 0.0090 | ***0.1357*** | 0.0042 | -0.0119 | ***-0.1392*** | -0.0079 |
| | *p* | 0.8160 | ***0.0005*** | 0.9125 | 0.7582 | ***0.0003*** | 0.8395 |
| *Rubus chamaemorus* | ***τ*** | -0.2086 | -0.0256 | -0.2496 | 0.1077 | -0.0459 | 0.2810 |
| | *p* | 0.0160 | 0.7682 | 0.0043 | 0.2136 | 0.5974 | 0.0013 |
